# The Relationship between Histological Composition and Metabolic Profile in Breast Tumors and Peritumoral Tissue Determined with ^1^H HR-MAS NMR Spectroscopy

**DOI:** 10.3390/cancers15041283

**Published:** 2023-02-17

**Authors:** Agnieszka Skorupa, Mateusz Ciszek, Maria Turska-d’Amico, Ewa Stobiecka, Ewa Chmielik, Ryszard Szumniak, Andrea d’Amico, Łukasz Boguszewicz, Maria Sokół

**Affiliations:** 1 Department of Medical Physics, Maria Skłodowska-Curie National Research Institute of Oncology, Gliwice Branch, 44-102 Gliwice, Poland; 2 The Oncological and Reconstructive Surgery Clinic, Maria Skłodowska-Curie National Research Institute of Oncology, Gliwice Branch, 44-102 Gliwice, Poland; 3 Tumor Pathology Department, Maria Skłodowska-Curie National Research Institute of Oncology, Gliwice Branch, 44-102 Gliwice, Poland; 4 Department of PET Diagnostics, Maria Skłodowska-Curie National Research Institute of Oncology, Gliwice Branch, 44-102 Gliwice, Poland

**Keywords:** metabolomics, NMR spectroscopy, breast cancer, heterogeneity, stroma, fibrosis

## Abstract

**Simple Summary:**

Breast cancer is the most prevalent malignancy all over the world. Intraoperative histological imaging of frozen tissue sections is frequently used in the evaluation of surgical margins for the presence of residual transformed cells. However, this method is time consuming, prone to sampling error and subjective interpretation. The purpose of this work was to investigate a sensitivity of the ^1^H HR-MAS NMR (high-resolution magic angle spinning nuclear magnetic resonance) technique in the detection of cancer cells in the tissue material collected during breast-conserving surgery and to gain an insight into a metabolic reprogramming of intratumoral fibrosis. The multivariate classification models permitted discrimination of the cancerous from non-cancerous samples with an accuracy of 87%. The accumulation of several metabolites (inter alia lactate, glutamate, succinate) in the fibrotic tissue within the tumor in reference to the extratumoral fibrous connective tissue was detected. The results of our work contribute to the increased understanding of breast cancer heterogeneity.

**Abstract:**

Breast tumors constitute the complex entities composed of cancer cells and stromal components. The compositional heterogeneity should be taken into account in bulk tissue metabolomics studies. The aim of this work was to find the relation between the histological content and ^1^H HR-MAS (high-resolution magic angle spinning nuclear magnetic resonance) metabolic profiles of the tissue samples excised from the breast tumors and the peritumoral areas in 39 patients diagnosed with invasive breast carcinoma. The total number of the histologically verified specimens was 140. The classification accuracy of the OPLS-DA (Orthogonal Partial Least Squares Discriminant Analysis) model differentiating the cancerous from non-involved samples was 87% (sensitivity of 72.2%, specificity of 92.3%). The metabolic contents of the epithelial and stromal compartments were determined from a linear regression analysis of the levels of the evaluated compounds against the cancer cell fraction in 39 samples composed mainly of cancer cells and intratumoral fibrosis. The correlation coefficients between the levels of several metabolites and a tumor purity were found to be dependent on the tumor grade (I vs II/III). The comparison of the levels of the metabolites in the intratumoral fibrosis (obtained from the extrapolation of the regression lines to 0% cancer content) to those levels in the fibrous connective tissue beyond the tumors revealed a profound metabolic reprogramming in the former tissue. The joint analysis of the metabolic profiles of the stromal and epithelial compartments in the breast tumors contributes to the increased understanding of breast cancer biology.

## 1. Introduction

Breast cancer is the most prevalent malignancy all over the world, with 2.3 million newly diagnosed patients in 2020 and 685,000 deaths [1]. Adherence to the population screening recommendations permits early detection of the disease in many patients and increased implementation of a breast-conserving technique for surgical removal of a tumor [2,3]. Unfortunately, a recurrence rate of 3–15% within 10 years has been reported, despite appropriate adjuvant therapy [4,5]. The treatment failure may be explained by the presence of residual cancer cells in the tissues beyond the surgical margin or by the existence of molecular alterations within a histologically normal tissue predisposing for tumor development [6,7]. The currently used clinical and pathological factors (such as histologic grade, hormone receptor status, human epidermal growth factor receptor 2 (HER2) status, tumor size, molecular subtype) are not sufficient to explain the diversity in treatment results [5]. Therefore, further molecular studies of the tumors and the peritumoral tissues are warranted.

Until recently, diagnosis and prognostication in breast cancer patients were mainly based on evaluation of the histological and molecular alterations in the transformed epithelial component of tumors. In response to the accumulating evidence on the important role of the tumor microenvironment (composed of stromal cells, immune cells, vasculature, extracellular matrix, and various secreted factors) in cancer genesis, growth and metastasis [8,9,10], the World Health Organization recommended histological assessment of the tumor milieu (in terms of tumor-infiltrating lymphocytes, fibrotic focus and Programmed Cell Death Protein 1/Programmed Cell Death Ligand 1 (PD-1/PD-L1)) expression for breast cancer classification [11]. Although prognostic relevance of the tumor-to-stroma ratio within tumors was observed in many works [12,13,14], the metabolic processes behind the cancer cell–stroma interactions are not well characterized.

Because cancer-associated fibroblasts (CAFs) are one of the dominant components of the breast tumor environment, metabolic cross-talk between these cells and transformed cells is a subject of an ongoing work [15,16]. Altered metabolism is considered to be a crucial driver of various fibrotic pathologies, beyond cancer [17]. Interestingly, perturbations in important metabolic pathways (such as glycolysis or glutaminolysis) are shared between the malignant and fibrotic cells [18].

NMR spectroscopy and mass spectrometry combined with multivariate analysis are helpful tools in metabolic profiling of tumors. This approach is known as metabolomics and has become a powerful approach to discover biomarkers, understand complex dis-ease processes and to characterize the differences between tumor and healthy tissues. In cancer, metabolomics detects oncological developments by evaluating measurable meta-bolic profiles and selecting metabolic pathways through global variations in metabolites [19,20]. Its standard workflow includes sample collection, data profiling, pattern recognition and final validation. Usually, metabolomics studies provide abundant biochemical information for further tumor research, aiming at effectively promoting the process of ear-ly diagnosis and therapy [21].

Spatially resolved methods enable various tissue types in the excised material to be classified and the tumor margins to be precisely delineated [22,23]. A possibility of detection of the metabolic differences between a tumor-associated stroma and an extratumoral one indicates that these techniques provide additional information beyond standard histopathology [22,24]. ^1^H HR-MAS NMR profiling of bulk tissues combined with a detailed histological characterization of the studied samples also permitted development of classification models enabling malignant cells recognition with an accuracy of 90% [25].

In this work, we exploited the non-destructive nature of the ^1^H HR-MAS NMR technique to find the relation between the histological content and the metabolic profiles of breast tumor and the adjacent normal breast tissue samples. Although the metabolic features of breast cancer (discrimination between cancerous and non-cancerous tissue [25], correlation to the known prognostic factors [26,27], response to the treatment [28,29,30], intratumoral heterogeneity [31,32,33]) have been studied in previous works using this method, the differences in metabolic status between the intratumoral tissue stroma (fibrosis) and the extratumoral one have not been characterized.

The purpose of this work was to investigate a sensitivity of ^1^H HR-MAS NMR technique in the detection of cancer cells in tissue material collected during breast-conserving surgery and to gain an insight into metabolic reprogramming of intratumoral fibrosis in breast cancer. 

## 2. Materials and Methods

### 2.1. Patients and Tissue Samples

This was a single center, prospective observational ex vivo ^1^H HR-MAS NMR study approved by the Ethics Committee at Lower Silesian Chamber of Physicians in Wrocław (resolution No. 29/11/2017) and informed consent was obtained from all patients. All research was performed in accordance with the relevant guidelines and regulations. The study group consisted of 39 patients with primary operable breast cancer (without neoadjuvant treatment), whose largest tumor dimension on radiological examination was less than 30 mm (cT_2_ N_0–1_ M_0_), consecutively scheduled for a surgery in National Research Institute of Oncology in Gliwice. To improve the reproducibility of the surgical technique, all operations were performed by the same team of two surgeons, using the same method, i.e., tumor resection and cavity shaving.

The patients were diagnosed with invasive ductal carcinoma (32 cases) and invasive lobular carcinoma (7 cases) with no lymph node involvement. Other clinico-pathological characteristics of the examined group (including patients’ age, tumor grade, hormone receptor status, HER2 status, T-classification) are presented in Table 1. The breast cancer subtypes were classified according to the immunohistochemistry results as: luminal A (ER- and/or PR-positive, HER2-negative, Ki-67 labeling index less than 14%), luminal B (HER2 negative): (ER- and/or PR-positive, HER2-negative and Ki-67 labeling index greater than or equal to 14%), luminal B (HER2 positive): (ER- and/or PR-positive and HER2-positive), HER2 type (ER- and PR-negative and HER2-positive) and TNBC type (ER-, PR- and HER2-negative) [34]. 

Along with a routine intraoperative histological assessment of the resected specimens, the multiple tissue samples from the tumor (T), the tumor border (TB), the tumor adjacent normal appearing tissue at three distances from the tumor border (less than 1 cm (N_d<1cm_), equal to 1 cm (N_d=1cm_), and above 1 cm (N_d>1cm_)) were collected. The weight of the samples excised from different areas was similar and amounted to 13.8 ± 2.8 mg (mean ± SD) for all specimens. The total number of collected samples was 156. The excised tissues were frozen in liquid nitrogen and stored at −80° until the ^1^H HR-MAS NMR measurements.

### 2.2. ^1^H HR-MAS NMR

The HR-MAS ^1^H NMR experiments were performed on a Bruker Avance III 400 MHz spectrometer (Bruker BioSpin GmbH, Karlsruhe, Germany) equipped with a ^1^H optimized 4-mm ^1^H/^13^C MAS probe. The frozen tissue samples were weighted and placed in 30 μL disposable Kel-F inserts filled with a 5 μL cold solution of 25 mM sodium formate in D_2_0 for the shimming and locking purposes. The inserts were introduced into 4-mm zirconium HR-MAS rotors. Because the analysis of NOESY spectra of breast tumors and normal breast tissue is complicated due to a considerable overlap of the signals originating from the lipid and small molecular weight metabolites, several spectral editing techniques were included in the measurement protocol. The Carr–Purcell–Meiboom–Gill (CPMG) spectra representative of small molecular weight metabolites were acquired using a cpmgpr1d sequence (a relaxation delay of 4 s, an effective echo time of 280 ms, a spectral width of 20 ppm, 65 k points, an acquisition time of 4.08 s and 256 scans). The slow-moving macromolecular compounds were highlighted in the diffusion edited spectra (DIFF) measured with the use of ledbpgp2s1dpr sequence (a relaxation delay of 4 s, Δ = 200 ms, δ = 2 × 2 ms, 64 scans, 65 k data points, a spectral width 30 ppm, an eddy current delay 5 ms, a sine-shaped gradient with 36 G/cm followed by a 200 μs delay for gradient recovery). The spectra were acquired at 4 °C (calibrated using methanol) with a magic angle spinning at 4000 Hz.

After, the ^1^H HR-MAS NMR measurements the specimens were frozen for histological evaluation of the tissue content. Before this evaluation, the samples were fixed in 10% formalin and embedded in paraffin. The samples were cut into multiple (2–5) 3 µm-thick cross-sections (at distances adjusted based on the sample amount) and stained with haematoxylin and eosine (H&E). The percentage fractions of the tumor tissue (including the cancer cells and stroma), cancer cells, intratumoral fibrotic stroma, necrosis, inflammation, glandular and extratumoral fatty tissue, as well as the fibrous connective tissue, were assessed by an experienced pathologist. The degree of fibrosis was scored according to the amount of collagen fibers and the number of fibroblasts: grade 1–small amount of collagen fibers, large number of fibroblasts; grade 2–intermediate between 1 and 3; grade 3–large number of collagen fibers and a low number of fibroblasts [35]. 

### 2.3. Data Preprocessing

The initial preprocessing of the spectra was performed in TopSpin 3.1 (Bruker BioSpin, Rheinstetten, Germany). The free induction decay signals (FIDs) were multiplied by an exponential function (line broadening of 1 Hz), subjected to a Fourier transformation, phase correction and linear baseline correction. The CPMG spectra were additionally subjected to multipoint baseline correction in the spectral region from 2.95 to 4.8 ppm in MestReNova 10 software (Mestrelab Research, Santiago, Spain). The chemical shifts were calibrated based on the formate peak at 8.44 ppm. The spectra were normalized using sample weight. 

The initial interrogation of the spectral data revealed a considerably lower signal-to -noise ratio for the small molecular weight compounds (CPMG spectra) in the samples from beyond the tumors’ locations as compared to the appropriate values obtained for the lesions spectra. Therefore, the analysis was focused on the spectral regions corresponding to the measurable signal intensities in the tumors. The regions representing the specific metabolites were carefully chosen to minimize the overlap with other compounds and were manually integrated in the whole dataset (the spectra acquired both from tumors and normal appearing tissue). Due to a substantial signal crowding in the spectral area from 3 to 3.3 ppm, the integral intensities corresponding to choline, phosphocholine and glycerophosphocholine were calculated from the line-fitted signals using MestReNova 10 software. An automatic deconvolution of the signal, assuming a generalized Lorentzian lines-shape, was performed for this purpose. The results are presented using the major metabolites contributing to the integrated spectral regions. These metabolites were identified using 2D HR-MAS NMR spectra (^1^H-^1^H J-resolved, ^1^H-^13^C HSQC, ^1^H-^1^H TOCSY) measured for selected samples, the published data [31] and publicly available databases [36]. However, when interpreting the results, the possible contributions from other, less abundant compounds to the integrated areas should be taken into account. Both major and minor metabolites corresponding to the integrated regions are listed in Table S1 (Supplementary Materials).

The metabolite levels (expressed as the signal integrals) obtained from the CPMG and DIFF spectra were concatenated in a single **X** data matrix and subjected to a multivariate analysis in SIMCA 17.0 software (Sartorius Stedim Biotech, Umea, Sweden). The analysis was preceded by automatic logarithmic transformation of selected variables (if required) and unit variance scaling.

### 2.4. Multivariate Data Analysis

#### 2.4.1. Unsupervised Principal Component Analysis (PCA)

**Figure 1:**
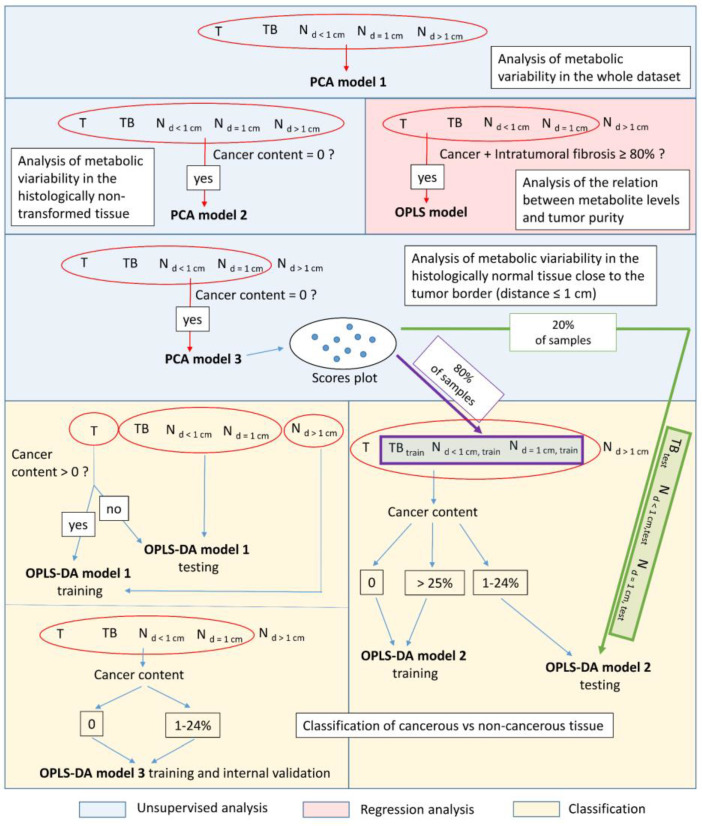
Multivariate analysis scheme.

The multivariate analysis was performed according to the scheme presented in Figure 1. The natural grouping of the samples according to their excision location (T, TB, N_d<1cm_, N_d=1cm_, N_d>1cm_) and the histologic content was investigated using PCA. This technique was exploited in the analysis of the whole dataset (PCA model 1), and in the analysis of the spectra collected from the histologically verified non-transformed tissue (PCA model 2). The exclusion of N_d>1cm_ samples from a further PCA modeling (PCA model 3) permitted visualization of the main variance sources within the histologically normal tissue at the close proximity to the tumor (at the distance ≤ 1 cm). Of note, based on the distribution of the samples in the scores plot resulting from the PCA model 3, the non-malignant tissue samples from the close tumor surroundings were divided into two subsets. One (involving 80% of the samples) was included in the training set for the Orthogonal Partial Least Squares Discriminant Analysis (OPLS-DA) model 2 construction, whereas the other (20% of the samples) was exploited for testing the predictive ability of this model (see the description of the OPLS-DA model 2). The criterion used for categorization of the samples to either the training or test sets was to obtain an even distribution of the specimens belonging to both groups on the projection plane formed by two first principal components in PCA model 3.

#### 2.4.2. Supervised Discrimination of Cancerous vs. Histologically Normal Tissue

The usefulness of ^1^H HR-MAS NMR method in classification of the malignant vs non-malignant tissue was evaluated based on the OPLS-DA model 1 constructed to differentiate the cancerous samples (obtained from a T location) from the histologically normal tissue at a distance above 1 cm from the tumor border (N_d>1cm_ location). The non-cancer specimens obtained from T location and the samples excised from the TB, N_d<1cm_ and N_d=1cm_ areas were used for the external validation of the model’s ability to detect the cancer cells.

Another approach used for assessment of the sensitivity of ^1^H HR-MAS NMR technique in identification of the transformed tissue component was the development of the OPLS-DA model 2, separating the samples collected from the T, TB, N_d<1cm_, N_d=1cm_ locations (characterized by the cancer purity ≥ 25%) from the histologically normal tissue excised from these areas. A total of 80% of the non-malignant tissue specimens collected at a distance ≤ 1 cm from a tumor border were used in the model training (TB_train_, N_d<1cm,train_, N_d=1cm,train_), whereas the remaining ones were included in the validation set (TB_test_, N_d<1cm,test_, N_d=1cm,test_). The categorization of the samples to the training or test sets was based on their distribution in the scores plot obtained from PCA model 3. The samples collected from the T, TB, N_d<1cm_, N_d=1cm_ locations characterized by the cancer cell percentage less than 25% were also used for model validation.

The minimum number of samples required to obtain reliable multivariate models differentiating cancer from normal tissue (OPLS-DA 1 and 2) was assessed retrospectively using the power analysis option of MetaboAnalyst 5.0 software [37].

Although an internal validation usually results in the over-optimistic results, the OPLS-DA model 3 was developed based on all available samples characterized by the cancer cells content < 25% and non-involved tissue from the close tumor surroundings (T, TB, TB, N_d<1cm_, N_d=1cm_ locations) and was tested using internal sevenfold cross validation.

#### 2.4.3. Orthogonal Partial Least Squares Regression (OPLS) Modeling of the Relation between the Cancer Content and the Spectral Integrals

The relationship between the cancer cell fraction (**Y** vector) and a metabolic profile (**X** data matrix) was analyzed using an OPLS regression method. The tissue samples collected from the T, TB, N_d<1cm_ and N_d=1cm_ locations containing mainly cancer cells and intratumoral fibrotic stroma (the total contribution of these two tissue components was required to be above 80%) were included in this analysis. 

The results of multivariate analysis are presented by means of the score and loading plots. The models were described by the fraction of the explained variation (R^2^) and the fraction of the variation predicted by sevenfold cross validation (Q^2^). The statistical significance of the created models was tested using cross validation analysis of variance (CV-ANOVA).

### 2.5. Comparison of the Metabolic Content of Cancer, Intratumoral Fibrotic Stroma and Extratumoral Connective Tissue–A Univariate Analysis

Linear regression analysis was exploited to examine the relationship between the metabolic levels (in terms of spectral integrals) and a tumor purity in the samples set analyzed using an OPLS regression (containing mainly cancer cells and intratumoral fibrotic stroma). The analyses were performed for the whole group of tumors and for two subgroups according to the histological grade (I vs. II/III). A homogeneity of the slopes test was used to evaluate the differences in the regression fits between the luminal A and luminal B subtypes. An extrapolation of the linear relationships to 0% cancer content was applied to find the metabolic levels of pure fibrotic stroma, whereas an extrapolation to 100 was applied to determine those levels in pure cancer tissue. The spectral integrals obtained for the abovementioned tissue components were compared with the values found for the histologically normal samples containing mainly extratumoral connective tissue (>80%). The differences between intra- and extratumoral stroma were considered significant when the confidence intervals (at the 95% level) of the metabolite levels predicted at 0% cancer content were found not to overlap with the confidence intervals of the mean metabolite levels in the connective tissue beyond a tumor. The non-overlapping confidence intervals at a level of 90% were indicative of the trends.

Because a contribution of fatty tissue in the samples excised from extratumoral stroma was higher than for the tumoral ones, the spectral integrals obtained for the specimens containing mainly extratumoral fibrous tissue were adjusted for a non-zero fraction of fat before the comparison to the values determined for intratumoral fibrosis. These adjustments were performed based on a linear regression analysis between the integral intensities in the evaluated spectral regions and fatty tissue content in the N_d=1cm_ and N_d>1cm_ locations. The results of this analysis are presented in Supplementary Materials (Tables S2 and S3). 

The differences between the grade I tumors and the grade II/III tumors were assessed based on the metabolites’ levels predicted at 60% cancer cells content.

## 3. Results

### 3.1. The Results of the Post-^1^H HR-MAS NMR Histopathology

The histopathological analysis of the samples previously examined using ^1^H HR-MAS NMR was possible for 90% of the collected specimens (140/156). The remaining samples [one sample excised from T area (invasive lobular carcinoma, grade 2, luminal B) and 15 samples excised from the areas beyond tumors] were damaged during retrieval from disposable inserts or histological preparation. Table 2 shows the obtained results. Cancer cells were found in 36 out of 38 tumor samples, in 8 out of 11 TB samples, in 9 of 26 samples collected from N_d<1cm_ and in 1 of 30 samples collected at N_d=1cm._ The samples from the T location contained mainly intratumoral fibrotic stroma (median: 70%) and cancer cells (median cancer purity: 30%). The median cancer purity in the samples from TB was of 3%. Moreover, these specimens contained substantial amounts of the extratumoral fatty and fibrous connective tissues; such tissue types were the main components of the material collected at the N_d<1cm_, N_d=1cm_ and N_d>1cm_ locations. As is revealed from Table 2, the percentage contribution of other tissue types (such as glandular tissue, inflammation and necrosis) is marginal in the evaluated tissue material.

To facilitate the comparison of the metabolic profiles, the samples most representative of cancer, intratumoral fibrosis, extratumoral fibrous connective tissue and fatty tissue were selected according to the criteria presented in Table 3.

Figure S1 shows the exemplary H&E-stained sections of the post-HR-MAS NMR specimens. Of note, the samples representing an extratumoral fibrous connective tissue were mainly assigned a fibrosis grade 1, whereas the intratumoral fibrosis samples–grades 2 and 3.

**Figure 2:**
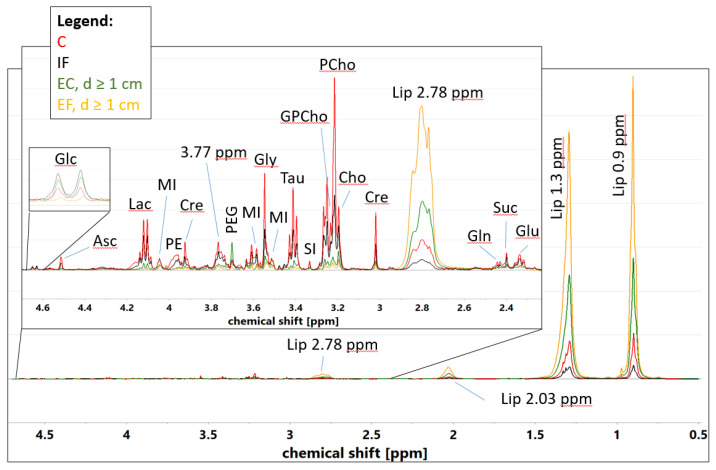
The average ^1^H CPMG HR-MAS NMR spectra calculated for the samples representative of cancer (C), intratumoral fibrosis (IF), extratumoral fatty tissue (EF, d ≥ 1 cm) and extratumoral fibrous connective tissue (EC, d ≥ 1 cm). The assigned low-molecular-weight signals: Glc–glucose, Asc–ascorbate, Lac–lactate, MI–myo-inositol, PE–phosphoethanolamine, Cre–creatine, 3.77 ppm–signal corresponding to *α*-H group of amino acids, PEG–polyethylene glycol (contaminant), Gly–glycine, Tau–taurine, SI–scyllo-inositol, GPCho–glycerophosphocholine, PCho–phosphocholine, Cho–choline, Glu–glutamate, Suc–succinate, Gln–glutamine. Residual lipid signals (Lip 0.9 ppm, Lip 1.3 ppm, Lip 2.03 ppm, Lip 2.78 ppm) are also visible.

Figure 2 shows the averaged ^1^H CPMG HR-MAS NMR spectra of the different tissue types. Obviously, the residual lipid signals at 0.9 ppm and 1.3 ppm predominate in these spectra. However, the regions from 2.9 to 4.7 ppm and from 2.2 to 2.6 ppm are free from a lipid contribution and, thus, the signals of low molecular weight metabolites are visible. For the samples with the highest content of neoplastic cells the signals are strongest, whereas for the samples containing mainly intratumoral fibrosis (with a fraction of neoplastic cells < 15%), the intensities of the signals are intermediate between those for the neoplastic and non-cancerous tissues (adipose and connective tissue). The only metabolite shown to be less abundant in the malignant samples in comparison to intratumoral fibrosis and extratumoral connective tissue is glucose. The spectral regions corresponding to the measurable signal intensities in tumors were integrated in the whole dataset and subjected to a multivariate analysis. The metabolites presenting the dominant and minor contributions to the evaluated spectra regions are given in Table S1 (Supplementary Materials).

### 3.2. Multivariate Analysis

#### 3.2.1. PCA Model 1

**Figure 3:**
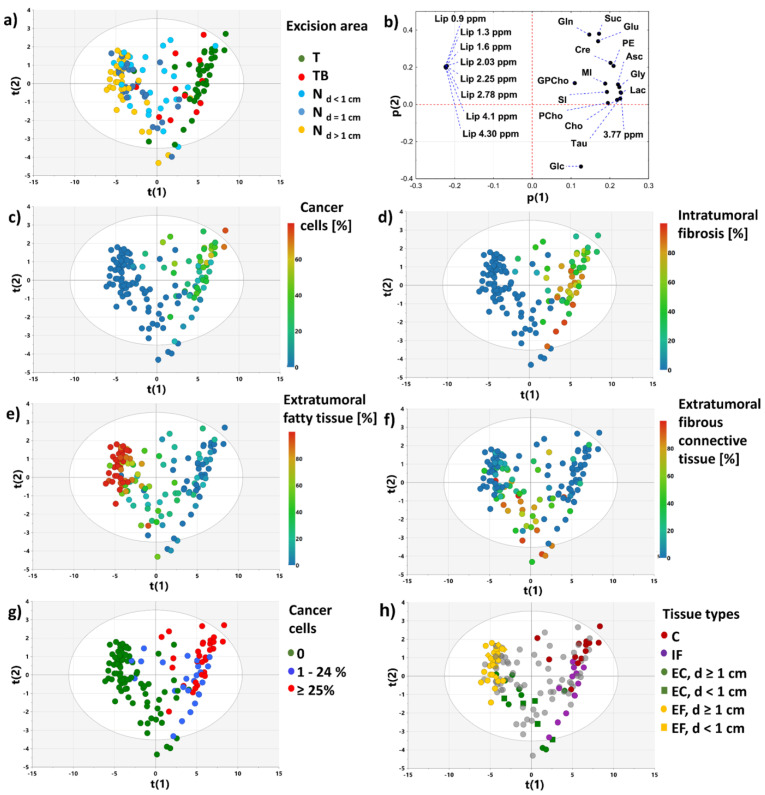
PCA model 1: the scores plot colored according to the excision area (**a**), the loadings plot (**b**), the scores plot colored according to the content of: cancer cells (**c**), intratumoral fibrosis (**d**), extratumoral fatty tissue (**e**), extratumoral fibrous connective tissue (**f**). The scores plot presenting the distribution of the non-transformed tissue samples, the malignant samples characterized by the cancer cell content below 25% and the malignant samples characterized by the cancer cell content greater than or equal to 25% (**g**). The scores plot presenting the distribution of the samples of specific tissue types (according to the criteria defined in Table 3): C–cancer; IF–intratumoral fibrosis; EC, d ≥ 1 cm–extratumoral fibrous connective tissue at a distance ≥1 cm from the tumor border; EC, d < 1 cm–extratumoral connective tissue at a distance <1 cm from the tumor border; EF, d ≥ 1 cm–extratumoral fatty tissue at a distance ≥1 cm from the tumor border; EF, d < 1 cm–extratumoral fatty tissue at a distance <1 cm from the tumor border (**h**). The scores for the i-th component are denoted as t(i), the loadings for the i-th component–as p(i). Abbreviations: Glc–glucose, Asc–ascorbate, Lac–lactate, MI–myo-inositol, PE–phosphoethanolamine, Cre–creatine, 3.77 ppm–signal corresponding to *α*-H group of amino acids, Gly–glycine, Tau–taurine, SI–scyllo-inositol, GPCho–glycerophosphocholine, PCho–phosphocholine, Cho–choline, Glu–glutamate, Suc–succinate, Gln–glutamine, residual lipid signals (Lip 0.9 ppm, Lip 1.3 ppm, Lip 2.03 ppm, Lip 2.78 ppm).

Figure 3 presents the score and loading plots for the first two principal components obtained from the PCA analysis of the whole dataset. The presented projection plane accounts for 83.4% of the total variability. The samples in the score plots were colored according to the specimen excision area (Figure 3a) and the tissue content (Figure 3c–g). As seen the positions of the samples reflect the histological results. This can be better appreciated when the samples of the specific tissue types (according to the criteria defined in Table 3) are marked in the plot (Figure 3h). The first quadrant is occupied by the cancer cells-containing samples: the increased integral intensities for all evaluated low molecular weight metabolites (except glucose) are responsible for the observed grouping (Figure 3b). The samples containing mainly extratumoral fatty tissue fall into the second quadrant, corresponding to the high lipid levels. The clustering of the connective tissue samples collected from the tumors’ surroundings is apparent in the third quadrant of the projection plane, whereas the fourth quadrant gathers the mixture of the samples characterized by a relatively low cancer cell fraction (<25%), a high intratumoral fibrosis content and the samples containing a significant amount of a connective tissue excised beyond the tumors. The samples from the fourth quadrant are characterized by the high glucose level. 

#### 3.2.2. PCA Models 2 and 3

**Figure 4:**
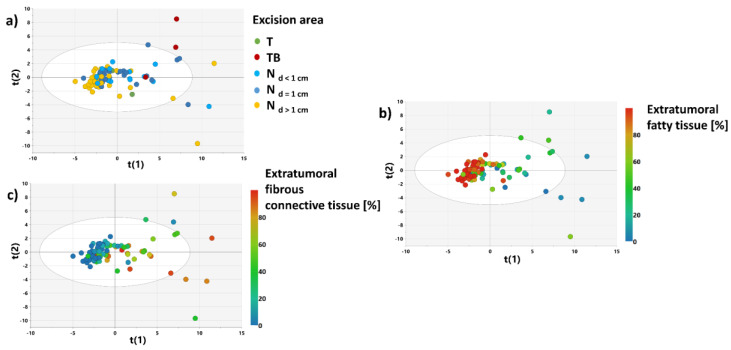
The scores plot obtained from the PCA model 2 colored according to: a sample excision area (**a**), a fatty tissue content (**b**), a connective fibrous tissue content (**c**). The scores for the i-th principal component are denoted as t(i).

Figure 4 shows the results obtained from the PCA model 2. The model was developed to examine the major sources of variance in the non-malignant tissue samples. The score plots for the first two principal components (representing 69.3% of the variation of the dataset) show that the distribution of the samples reflects a histological composition of the evaluated specimens rather than an excision area. The samples containing mainly a fatty tissue were assigned the negative scores for the first principal component, whereas the samples characterized by a higher contribution of a connective tissue were assigned the positive scores.

**Figure 5:**
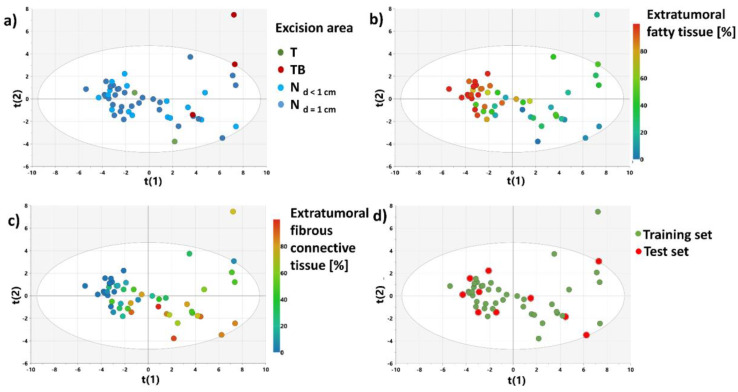
The scores plot obtained from the PCA model 3 colored according to: a sample excision area (**a**), the contents of fatty tissue (**b**) and connective tissue (**c**); a sample splitting into the test and training sets used for the OPLS-DA 2 model development and evaluation (**d**). The scores for the i-th principal component are denoted as t(i).

The PCA model 3 constructed after excluding the samples from a distance higher than 1 cm from the tumor border (Figure 5) provides similar conclusions. Based on the distribution of the samples in the scores plot, the specimen set was split into the training and test sets for construction and evaluation of the OPLS-DA model 2 (Figure 5d).

#### 3.2.3. OPLS-DA Model 1

**Figure 6:**
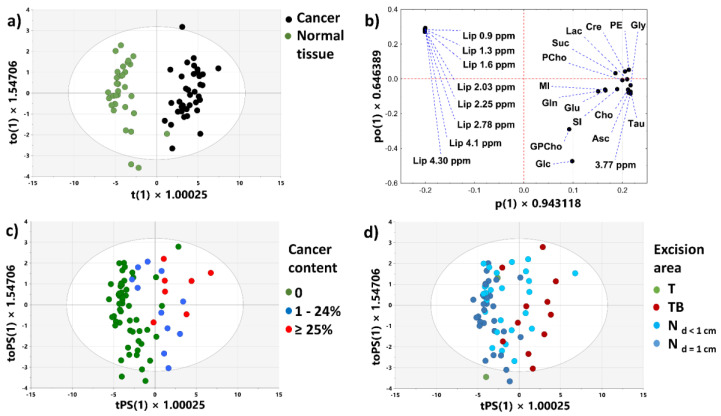
The OPLS-DA model 1: the scores plot (**a**), the loadings plot (**b**), the predicted scores plot colored according to the cancer content (**c**), the predicted scores plot colored according to the excision area (**d**). Scores and loadings for the predictive component are denoted as t(1) and p(1), whereas those for the orthogonal one—as to(1) and po(1). The predicted scores for the predictive component are denoted as tPS(1), whereas those for the orthogonal one—as toPS(1).

Figure 6a shows the score plots obtained from the OPLS-DA model 1 (1 predictive + 1 orthogonal latent variables, R^2^X = 81.6%, R^2^Y = 90%, Q^2^ = 86.1%, CV-ANOVA *p* value = 3.66 × 10^−27^) constructed to differentiate the cancerous and normal samples obtained from the T and N_d>1cm_ areas. The excellent separation between the groups is evident. The increased levels of all assessed metabolites (except glucose and glycerophosphocholine) and the low lipid levels in the malignant tissue contribute to this separation (Figure 6b). The predicted scores plot for the samples included in the external test set (two non-cancer samples excised from the T areas and the samples obtained from the TB, N_d<1cm_ and N_d=1cm_ locations) also shows a considerable grouping between the specimens containing cancer cells and the non-transformed tissue (Figure 6c). Of note, the TB samples are separated from the samples obtained from the N_d=1cm_ location in this plot. Assuming that the malignant tissue samples are assigned the positive PC1 scores, whereas the non-transformed tissue are assigned the negative ones, a classification accuracy of the model is 87% (sensitivity 72.2%, specificity 92.3%). Four of five misclassified cancerous samples were verified to contain a considerable amount of the extratumoral fatty tissue (>50%). The predicted scores plot colored according to the tissue contents is shown in the Supplementary Materials (Figure S2). The results obtained from the retrospective power analysis (Figure S3, Supplementary Materials) indicate that the power of 0.8 is achieved for a minimum sample size per group of 20 (a false discovery rate, FDR-adjusted *p*-value of 0.001). Thus, the number of specimens analyzed in our study is sufficient to obtain a meaningful multivariate classification model.

#### 3.2.4. OPLS-DA Model 2

**Figure 7:**
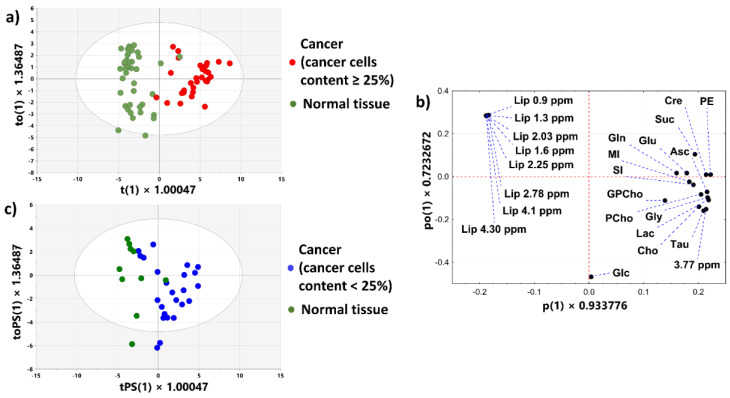
The OPLS-DA model 2: the scores plot (**a**), the loadings plot (**b**), the predicted scores plot (**c**). Scores and loadings for the predictive component are denoted as t(1) and p(1), whereas those for the orthogonal one—as to(1) and po(1). The predicted scores for the predictive component are denoted as tPS(1), whereas those for the orthogonal one—as toPS(1).

The OPLS-DA model 2 (Figure 7; 1 predictive + 1 orthogonal latent variable, R^2^X = 84.6%, R^2^Y = 82.3%, Q^2^ = 80%, CV-ANOVA *p* value = 2.55 × 10^−22^) was developed to separate the samples characterized by the cancer cells content ≥ 25% and the non-cancerous tissue from the tumor surroundings (at the distance less than or equal to 1 cm). The score and loading plots show that the increased levels of all metabolites (except glucose) and the low lipid levels in the malignant samples are responsible for the group’s separation along the first latent variable (Figure 7a,b). The predicted score plots for the external dataset composed of the specimens of the cancer purity 1–24% and 10 non-malignant tissue samples obtained from the close tumor proximity is shown in Figure 7c. The classification accuracy of the model is 76.5% (sensitivity 70.8%, specificity 90%). The predicted score plots colored according to the tissue content are shown in the Supplementary Materials (Figure S4). The results of the power analysis confirm that the sample size in the OPLS-DA model 2 is sufficient (Figure S5, Supplementary Materials).

#### 3.2.5. OPLS-DA Model 3

Results obtained from the OPLS-DA model 3 (1 predictive + 1 orthogonal latent variable, R^2^X = 73.6%, R^2^Y = 65.8%, Q^2^ = 58.6%, CV-ANOVA *p* value = 8.02 ×10^−13^) are presented in Figure S6 (Supplementary Materials). The cross-validated scores plot shows a considerable separation between the cancerous and non-involved tissue along the first latent variable. Of note, several normal tissue samples located at the border between these two groups are characterized by the relatively high content of glandular tissue (10–20%). It seems that a detailed metabolic characterization of all tumor tissue components could contribute to increased accuracy of discrimination between the samples characterized by the low cancer cells content and the non-transformed tissue.

#### 3.2.6. OPLS Model

**Figure 8:**
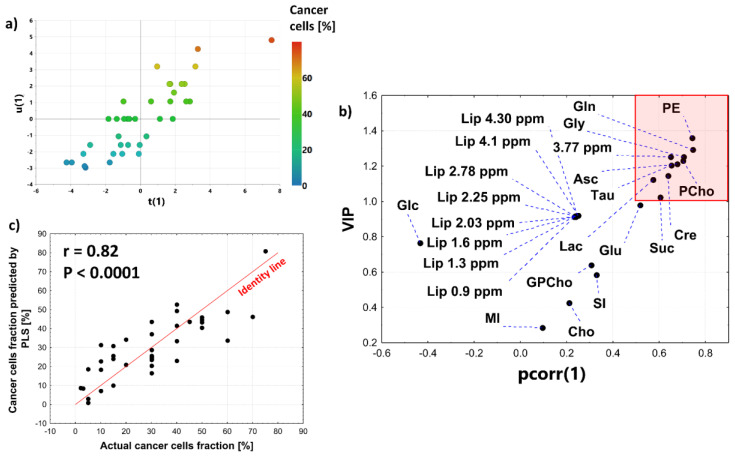
The OPLS model constructed to find the relationship between the metabolite levels (X) and a cancer purity (Y) in the samples containing mainly intratumoral fibrosis and cancer cells: (**a**) the u(1) vs. t(1) scores plot, (**b**) the pcorr(1) vs. VIP plot (the red shaded region corresponds to the VIP values  >  1 and pcorr(1) >  0.5) (**c**) the cancer cells content predicted by the model (based on sevenfold cross-validation) vs. the actual cancer cell fraction. The scores for the first component summarizing X variables are denoted as t(1), whereas those summarizing Y variables—as u(1).

Figure 8a presents the t(1) vs. u(1) scores plot obtained from the OPLS model (1 predictive + 1 orthogonal latent variable, R^2^X = 67.2%, R^2^Y = 73.6%, Q^2^ = 67.4%, CV-ANOVA *p* value = 5.9 × 10^−8^) constructed to find a relationship between a tumor’s purity and the metabolite levels for the samples containing mainly cancer cells and intratumoral fibrosis. The scatterplot presenting the loadings for the predictive latent variable scaled as correlations [pcorr(1)] vs Variable Importance at Projection (VIP) scores indicates that the cancer cell fraction is positively correlated to the levels of phosphoethanolamine, phosphocholine, glutamine, glycine, lactate, ascorbate, creatine, taurine and succinate (Figure 8b). These metabolites were described by pcorr(1) > 0.5 and VIP > 1. Figure 8c shows that the histologically verified percentage content of the transformed cells and the tumor purity predicted by the model (using sevenfold cross-validation) are in a general agreement (r = 0.82, *p* < 0.0001).

### 3.3. Univariate Linear Regression Analysis

**Figure 9:**
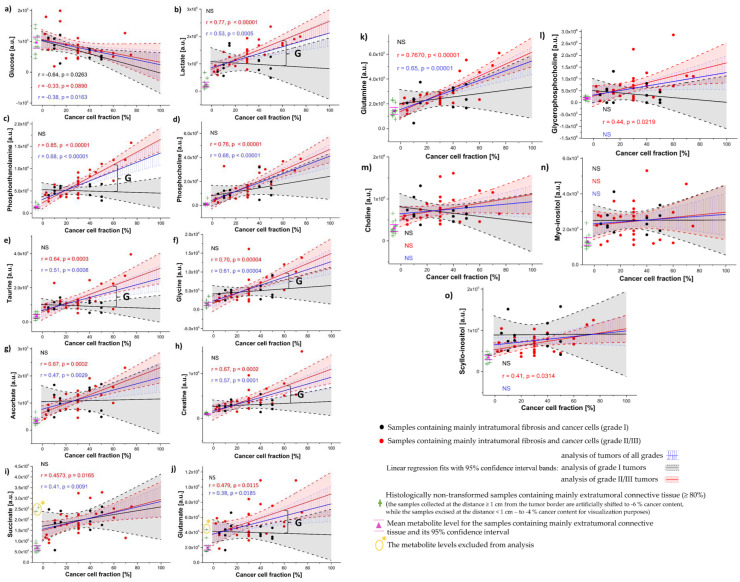
The relationships between glucose (**a**), lactate (**b**), phosphoethanolamine (**c**), phosphocholine (**d**), taurine (**e**), glycine (**f**), ascorbate (**g**), creatine (**h**), succinate (**i**), glutamate (**j**), glutamine (**k**), glycerophosphocholine (**l**), choline (**m**), myo-inositol (**n**), scyllo-inositol (**o**) and a cancer cell fraction. The linear fits (blue solid lines—the analysis of the tumors of all grades, black solid lines—the analysis of the grade I tumors, red solid lines—the analysis of the grade II/III tumors) with 95% confidence bands (blue dashed lines—the analysis of the tumors of all grades, black dashed lines—the analysis of the grade I tumors, red dashed lines—the analysis of the grade II/III tumors) were calculated for the samples containing mainly intratumoral fibrosis and cancer cells. Pearson correlation coefficients (r) between metabolite levels and cancer cell fraction are highlighted in blue (for tumors of all grades), black (for grade I tumors) and red (for grade II/III tumors). The levels of the metabolites corresponding to pure intratumoral fibrosis and pure cancer are determined from the extrapolation of the regression lines to 0% and 100% cancer content. Additionally, the metabolite levels measured in the samples representative of extratumoral fibrous connective tissue (green crosses) are presented. The pink triangle and pink lines indicate the mean metabolite levels obtained for these samples and their 95% confidence intervals. The significant differences between the grade I tumors and the grade II/III tumors are marked with a curly bracket (and a G letter) at a cancer cell fraction of 60%. Two outlying integral intensities in the spectral region corresponding to succinate (marked with a yellow asterisk) were not included in the calculation of the mean level of this metabolite in the fibrous connective tissue beyond tumor. A prominent singlet at 3.70 ppm assigned to polyethylene glycol (a component of optimal cutting temperature (OCT) fixative) was detected in ^1^H HR-MAS NMR spectra acquired from the samples exhibiting the extraordinarily high succinate signals. An outlying signal intensity in the spectral region corresponding to glutamate was also present in one of these samples and was excluded from the analysis. NS—not significant.

The relations between the metabolite levels and a tumor purity were also examined using a univariate linear regression. The results obtained from the analysis of tumors of all grades (containing mainly cancer cells and intratumoral fibrosis) and the results derived from the separate regression analyses for the low-grade (I) tumors and the higher-grade (II/III) ones are presented in Figure 9. One observation (the ascorbate level in a sample with the cancer cell fraction of 75%) was excluded from the analyses due to the extraordinarily high standardized residual (equal to 4.5) and Cook’s distance (equal to 2.6). The moderate positive correlations between several metabolites (phosphocholine, phosphoethanolamine, lactate, glycine, glutamine, creatine and taurine) and a cancer cell content (0.5 < r < 0.7) and the weaker positive associations between ascorbate, succinate and glutamate and a tumor purity (0.3 < r < 0.5) were found based on the analysis of the tumors of all grades. Glucose was found to be the only metabolite negatively related to a cancer cells percentage (r = −0.38, *p* < 0.05) in this analysis.

A separate regression analysis for the higher-grade (II/III) tumors revealed strong positive associations between a tumor purity and the levels of phosphocholine, lactate, phosphoethanolamine, glycine and glutamine (r > 0.7). The moderate positive correlations between a cancer content and the levels of ascorbate, taurine and creatine were also found (0.5 < r < 0.7) in this group of tumors whereas glutamate, succinate, scyllo-inositol and glycerophospcholine were observed to be weakly correlated (0.3 < r < 0.5) to a tumor purity. Of note, the evaluated metabolite levels (except glucose) were not observed to be significantly associated with a cancer content in the grade I tumors. The moderate negative relation between the glucose level and a tumor purity was detected in this group of tumors (r = −0.64, p < 0.05). 

Apart from the linear fitting results, the metabolite levels extrapolated to 0% and to 100% cancer cell content corresponding, respectively, to pure intratumoral fibrosis and pure cancer tissue are presented in Figure 9. The levels measured in a histologically normal fibrous connective tissue excised at the N_d<1cm_ and N_d≥1cm_ locations are also shown. Intratumoral fibrotic stroma is characterized by higher lactate, succinate, glutamate, phosphoethanolamine, myo-inositol, scyllo-inositol, choline and ascorbate than extratumoral connective tissue, as revealed by the analysis of the tumors of all grades. Whereas a stromal accumulation of lactate, succinate and glutamate is also visible in the separate analyses of both low-grade (I) and higher-grade (II and III) tumors, ascorbate, choline, myo-inositol, and scyllo-inositol were found to be increased in the fibrotic tissue in the grade I tumors and trended towards an increase in the higher-grade (II/III) ones (Supplementary Materials, Figure S7). The 90% confidence intervals for the contents of the latter metabolites predicted at 0% cancer cells percentage in the grade (II/III) tumors were observed not to overlap with the confidence intervals for the mean contents of these compounds in the extratumoral tissue. Additionally, glycine, phosphoethanolamine, creatine and taurine accumulation (relative to the connective tissue beyond tumor) was observed in the stroma of grade I cancer only.

The metabolic changes associated with tumor malignancy were revealed by predicting the metabolite levels at 60% cancer cell fraction, with higher levels of lactate, taurine, phosphoethanolamine, glycine, glutamate, and creatine observed in grade II/III tumors compared to grade I tumors.

**Figure 10:**
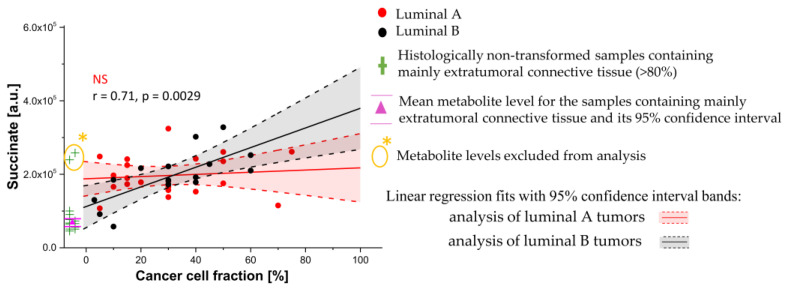
The relationships between a succinate level and a cancer cell fraction in the luminal A and luminal B subgroups. The linear fits (red solid lines—the analysis of luminal A tumors, black solid lines—the analysis of luminal B tumors) with 95% confidence bands (red dashed lines—the analysis of luminal A tumors, black dashed lines—the analysis of luminal B tumors) were calculated for the samples containing mainly intratumoral fibrosis and cancer cells. Pearson correlation coefficients (r) between a succinate level and a cancer cell fraction in luminal B tumors is highlighted in black. The relation for luminal B tumors was not significant (NS, highlighted in red).

Regression analyses were performed separately to examine the relationship between metabolite levels and cancer cell fraction in the luminal A and luminal B subgroups. The homogeneity of slopes test revealed that the correlation between succinate and a tumor purity depends on the intrinsic molecular subtype (*p* < 0.05). Whereas no association was observed for the luminal A tumors, a moderate positive correlation (r = 0.71, *p* = 0.0029) was detected for the luminal B ones (Figure 10). Interestingly, a stromal accumulation of succinate was visible only for the luminal A tumors.

## 4. Discussion

The aim of breast-conserving surgery (BCS) is a complete resection of tumor with a surrounding rim of healthy tissue while preserving the natural appearance of the breast. Intraoperative histological imaging of frozen tissue sections is frequently used in evaluation of the surgical margins for the presence of residual cancer. However, this method is time consuming, prone to sampling error and subjective interpretation. In the last decade, feasibility of metabolomics based on mass spectrometry and ^1^H HR-MAS NMR spectroscopy in a real-time detection of cancer cells in the surgical specimens was shown [25,38].

Bathen et al. (in their ^1^H HR-MAS NMR study of 328 samples from 228 breast cancer patients) reported that the accuracy of discrimination between normal and cancerous breast tissue is around 90% based on internal double leave-20%-out cross-validation [25]. The accuracy of the OPLS-DA 1 model (constructed to differentiate the cancerous tissue excised from tumors and normal tissue at a distance above 1 cm from the tumor border) is close to this value according to external validation (using the samples excised from the close tumor surroundings at a distance ≤ 1 cm from a tumor border). Given that most of the misclassified cancerous samples were characterized by the low cancer purity (Figure 6c), we tried to better characterize the performance of the method for such samples. The OPLS-DA model 2 was developed to discriminate the specimens characterized by a higher cancer content (≥25%) and noninvolved tissue from the tumor surroundings (at a distance ≤ 1 cm), whereas the samples characterized by the cancer purity < 25% were used for external validation of this model. The estimated accuracy amounted to 76.5% using this approach. Results obtained from OPLS-DA 3 model indicate that detailed analysis of metabolic differences between the glandular and cancerous tissue could contribute to the increased accuracy of detection of the transformed cells in postoperative specimens.

Although a cancer cell fraction has been reportedly shown to constitute the main source of variability in the ^1^H HR-MAS NMR spectra from breast tumors [25,27], the distribution of the samples according to the content of fat, intratumoral fibrosis and extratumoral fibrous connective tissue in the classification planes (obtained using pattern recognition analysis) has not been presented so far.

The unsupervised PCA analysis performed in our work revealed that the samples representative of various tissue types occupy different regions of the principal component space. The up-regulation of all evaluated small molecular compounds (except glucose) and the reduced lipid levels were found to be responsible for the differentiation between the cancer and non-cancer samples. As revealed from the early studies of the tissue extracts the metabolic profiles of the cancerous breast tissue and the noninvolved, normal tissue from the surrounding areas differ significantly [39]. The characteristic features of the tumor extracts were the high concentrations of lactate, taurine and succinate, the increased concentration of phosphocholine and a very low phosphocreatine. In the spectra of the non-involved tissue, the signals from glucose and other carbohydrates were high, whereas most of the tumors had very low or no detectable levels of glucose [39]. This agrees with our observations of less abundant glucose in the malignant tissue in comparison to intratumoral fibrosis and extratumoral connective tissue, and of the higher presence of succinate, lactate, taurine and phosphocholine in the cancerous lesions. Thus, it may be concluded that both NMR methods provide substantially similar information for the metabolites being important for the diagnosis. The decreased levels of many small molecular weight compounds in the non-involved tissue samples compared to the cancerous ones can be explained by the lower density of the cells containing water soluble compounds in the normal breast tissue composed mainly of adipose and fibrous connective tissue. The more intense metabolic profile is expected for glandular tissue. However, the contribution of this component in the studied samples rarely exceeds 20% in the published works [25] (and in our data).

Interestingly, in their early studies of breast cancer metabolism, Sitter et al. reported a higher signal-to-noise ratio in the ^1^H HR-MAS NMR spectra from the “non-cancerous” samples (excised from the tumors but histologically confirmed to be free of tumor cells) than for the samples obtained from an adjacent healthy tissue [40]. The explanation for this finding was that tumoral samples had a higher fraction of connective and glandular tissue than normal breast samples, which were mainly composed of adipose tissue. However, this finding was not given much attention in future works. With increasing knowledge of the role of tumor stroma in tumor progression and metastasis [8,9,10], the detailed analysis of the “non-cancerous” tumor samples may contribute to a better understanding of the biology of breast tumors. The role of the metabolic properties of tumor microenvironment in the natural grouping of the ^1^H HR-MAS NMR spectra of breast tumors was underscored in a more recent study by Haukaas et al. [41]. The three metabolic clusters identified in this work differed significantly in the expression of the genes related to the collagen and extracellular matrix properties. Interestingly, the detected grouping was not associated with the traditionally used intrinsic genetic PAM50 subtypes (luminal A, luminal B, (HER2)-enriched, basal-like and normal-like), but could be related to the subtypes defined by a reverse phase protein array (RPPA), including four groups similar to the genetic variants (Basal, HER2, luminal A, and luminal A/B groups) and two groups (reactive I and reactive II) characterized by a higher expression of proteins in the tumor milieu.

A high compositional variability of breast tumors makes the quantitation of the ^1^H HR-MAS NMR spectra a non-trivial task [42]. The normalization to the signal mean or to the total area under the spectrum is expected to remove a high portion of variance associated with a tumor purity from the datasets. Indeed, the analysis of the mean normalized metabolite levels revealed only weak positive correlations between glycine and a cancer percentage and weak negative associations of myo-inositol and taurine to cancer content [43]. No significant dependence of the metabolite levels on a tumor purity in hormone receptor-positive cancer and triple-negative cancer was detected based on HR-MAS NMR data normalized to the total intensity by Choi et al. [33]. They also did not detect any metabolic differences between the tumors of different pathological grades.

The spectra scaling with respect to a sample weight is well suited for a direct analysis of the relation between a metabolite abundance and a sample composition. Using this method in our work, the levels of the evaluated compounds in pure stroma and in pure cancer tissue were inferred from the linear relationships between a metabolite content and a cancer percentage. A similar approach was used to determine the metabolic genes and pathways deregulated in cancer cells and tumor stroma in 20 tumor types [44]. Because breast cancer belongs to desmoplastic lesions, the main attention in the analysis was paid to the samples composed mainly of cancer cells and intratumoral fibrosis. An initial interrogation of the data revealed a grade (I vs. II/III) dependency of the correlation coefficient between the levels of the evaluated compounds and the cancer cell fractions in these samples. Whereas a glucose decrease as a function of a tumor purity was found for the grade I tumors, the positive associations of a tumor content to ascorbate, lactate, phosphoethanolamine, phosphocholine, glycine, taurine, creatine, glutamine, glutamate, glycerophosphocholine and succinate were detected for the higher-grade (II/III) lesions. The positive correlations of glycine, phosphocholine and glycerophosphocholine to a cancer percentage were reported by Sitter et al. in their analysis of the ERETIC-scaled metabolite levels (determined per gram tissue) in a heterogeneous group of patients of various prognoses [27]. Importantly, our analysis proved that the narrowing of the studied tumors group down to the higher-grades (II/III) revealed the stronger associations between the metabolite levels and cancer content than those observed in the joint analysis of the tumors of all grades. Thus, the variability in the ^1^H MRS MAS NMR spectra reflects a clinically important metabolic heterogeneity rather than the technical noise. A comparison of the metabolite levels predicted at 60% cancer cell fraction between the low (I) and higher (II/III) grade tumors revealed the metabolic changes associated with tumor malignancy: in the grade II/III tumors lactate, taurine, phosphoethanolamine, glycine, glutamate and creatine are higher than in the grade I ones.

Glucose is the primary substrate for energy production which is metabolized to pyruvate through glycolysis pathway in the cell cytoplasm. In aerobic conditions, pyruvate is decaroboxylated to Acetyl-CoA that fuels the mitochondrial tricarboxylic acid (TCA) cycle and oxidative phosphorylation (OXPHOS). Under the oxygen shortage or in the cells lacking functional mitochondria, pyruvate is reduced to lactate via lactate dehydrogenase. Although this process is less energetically efficient than mitochondrial respiration, it is faster [45]. An increased uptake of glucose and preferential fermentation of this metabolite to lactate is detected in cancer cells even in the presence of oxygen (Warburg effect). The glycolytic phenotype correlates with a breast cancer aggressiveness [46]. Ravazoula et al. found that the most important glucose transporter GLUT1 is expressed in 28% of grade 1 tumors, in 63.8% of grade 2 tumors and in 58.7% of grade 3 tumors [47]. A significant association between a high GLUT1 expression and a histological grade in breast cancer was also confirmed by Krzeslak et al. [48]. Our observation of elevated lactate level in the poorly differentiated tumors (grades II/III) in comparison to the grade I lesions indicates an enhanced aerobic glycolysis in the former ones. A fast rate of a Warburg pathway could also be responsible for the detected glycine accumulation in the high-grade (II/III) tumors. This metabolite is formed from serine through the serine hydroxymethyltransferase (SHMT) enzyme involved in glycolysis-related serine and glycine metabolism. The expression of this enzyme was shown to be dependent on the degree of a pathomorphological differentiation in breast cancer [49]. Although the immunohistochemical staining indicates that glycolysis and serine/glycine pathway in breast tumors are correlated [50], it is also possible that increased glycine in the high-grade tumors results from a deregulated choline metabolism. It is worth noting that choline can be either phosphorylated via choline kinase to form phosphocholine (Kennedy pathway) or can be oxidized to betaine which can then be demethylated to glycine. A metabolic shift from the production of phospholipids to the formation of glycine was observed in basal-like breast cancer xenografts in reference to the luminal-like ones [51]. The high glycine level in the clinical breast tumor samples was found to be a marker of a poor prognosis and a short survival [52]. Taking the other route of choline metabolism into account, the increased activity of choline kinase was observed in the higher-grade tumors and was considered to be a marker of malignancy [53]. In agreement with these findings, an elevated phosphocholine content in the high-grade tumors relative to the low-grade tumors was detected in a study by Beckonert et al. [54], whereas the increased ratios of phosphocholine to choline and phosphocholine to creatine were detected in the studies by Choi et al. [26] and Cheng et al. [55]. Elevated phosphocholine was suggested as a potential biomarker in identifying the resection margins [25]. Although our analyses revealed a higher level of phosphocholine in the grade II/III tumors than in the grade I ones, the difference was not found to be significant.

Apart from the changes in choline metabolism, the poorly differentiated tumors were also characterized by the increased phosphoethanolamine level in the study by Beckonert et al. [54]. Such metabolic change was also apparent in our work. The accumulation of phosphoethanolamine in breast tumors is related to the increased activity of ethanolamine kinase 1 enzyme [56]. However, under stress conditions (including glutamine deprivation), an elevated level of this metabolite was shown to be associated with Phosphate Cytidylyltransferase 2 (PCYT2) downregulation [57]. Interestingly, up-regulation in the levels of phosphoethanolamine via the latter metabolic route permitted cancer growth under nutrient starvation and was correlated to a poor prognosis in breast cancer [57].

An increased glucose utilization is often not sufficient to meet the high energy and biosynthetic requirements of proliferating cells. The most abundant amino acid in the body, glutamine is an important nutrient for replenishment of the TCA cycle, synthesis of macromolecules and glutathione. Whereas the basal-type breast cancer cells are known to be glutamine-dependent, luminal cells are more glutamine independent due to the expression of glutamine synthetase [58]. The conversion of glutamine to glutamate (the first step of glutaminolysis) is catalyzed via mitochondrial enzyme glutaminase (GLS). The positive expression of GLS1 was observed in 70% tumors by Choi et al. (regardless of the subtype) [46] and was correlated to a histological grade in canine mammary tumors [59]. The increased glutamate levels in the poorly differentiated tumors than in the grade I ones (as observed in our work) could be associated with these findings. Interestingly, recent studies underscore the important role of glutaminase in the aggressive subgroup of luminal breast cancer and in progression from luminal DCIS to invasive cancer [60].

Creatine is another compound found to be more abundant in the higher-grade (II/III) tumors than in the lower grade (I) ones. This metabolite participates in ATP production pathways and exhibits antioxidant capabilities [61]. Aberrant expression of creatine metabolism related proteins was observed in many tumor types, including breast cancer [62]. Specifically, the expressions of Slc6a8 (solute carrier family 6 member 8), which encodes the creatine transporter was reported to be associated with breast cancer grade in TNBC patients [61]. 

Through examination the relationships between the levels of the evaluated compounds and tumor purity, we were able to determine the metabolic composition of the pure intratumoral fibrotic stroma in the breast tumor samples we studied. The higher levels of lactate, succinate, glutamate, phosphoethanolamine, myo-inositol, scyllo-inositol, choline and ascorbate in this compartment than in the extratumoral fibrous connective tissue were revealed in the analysis of the tumors of all grades. Whereas a stromal accumulation of lactate, succinate and glutamate was visible in the separate analyses of both low-grade (I) and higher-grade (II and III) tumors, ascorbate, choline, myo-inositol and scyllo-inositol were found to be increased in the fibrotic tissue in the grade I tumors, revealing only a tendency to increase in the higher-grade ones. Additionally, the accumulations of glycine, phosphoethanolamine, creatine and taurine were observed in the stroma of the grade I cancer only.

The Warburg effect was initially considered a hallmark of cancer cells resulting from dysfunctional mitochondria. However, recent studies indicate that OXPHOS is one the most up-regulated metabolic pathways in malignant cells, whereas the intensity of aerobic glycolysis is comparable between the stromal and transformed compartments [44]. According to “the autophagic tumor stroma model of cancer” malignant cells induce a down-regulation of caveolin-1 in CAFs leading to oxidative stress, autophagy, mitochondrial breakdown and a metabolic shift to aerobic glycolysis in these cells [63]. Lactate secreted by CAFs to the tumor microenvironment is taken up by cancer cells, converted to pyruvate via lactate hydrogenase and used as a fuel for the TCA cycle and OXPHOS (the reverse Warburg effect). However, this theory does not universally describe all cancer-stroma interactions in breast tumors. Based on the glycolytic status of the cancer cells and tumor stroma (characterized by the expression levels of glucose transporter-1 protein (GLUT-1) and carbonic anhydrase IX (CAIX)), Choi et al. classified 740 samples of breast tumors into four metabolic phenotypes [46]. A total of 52% of the lower grade (I and II) tumors were characterized by a null phenotype (non-glycolytic cancer cells and stroma), whereas 34%—by a Warburg phenotype (glycolytic stroma, non-glycolytic cancer). A total of 51% of the high-grade (III) tumors exhibited the Warburg, 27%—null and 17%—mixed (glycolytic stroma, glycolytic cancer) phenotypes. The reverse Warburg type (glycolytic stroma, non-glycolytic cancer) was found in 54 of 740 breast tumors (7.3%). The low-grade luminal A tumors constituted the vast portion of the samples belonging to this group. Our findings suggest that grade I tumors do not exhibit an association between the cancer cell fraction and lactate levels, whereas lactate accumulation is observed in the stroma. These observations may indicate the NMR-visible trace of the reverse Warburg effect. The higher-grade (II/III) breast tumors were characterized by an elevated lactate level in the intratumoral stroma compared to the healthy tissue, and a positive correlation between the level of this metabolite and the cancer cell fraction related to a more aggressive behavior. Interestingly, Choi et al. found that grade III tumors accounted for a high proportion of the tumors classified as a mixed phenotype [46]. Moreover, elevated lactate in the intratumoral stroma presumably relates also to the transfer of this metabolite between the hypoxic, glycolytic cancer cells and the oxidative ones within the tumor milieu (metabolic symbiosis) [64].

Succinate is also more abundant in the intratumoral fibrosis than in the normal fibrous connective tissue. This important intermediate of the TCA cycle is accumulated in several tumor types due to the inactivating mutations in succinate dehydrogenase (SDH) [65]. However, the loss of the SDH genes (SDHA or SDHB) is reported in only 3% of breast cancers [66]. Whereas more than 50% of breast tumors exhibited 50% expression of this enzyme in the epithelial compartment, it was rarely detected in stroma [66]. Of note, increased succinate was observed in CAFs isolated from human colon cancer and melanoma and was related to the isocitrate dehydrogenase 3α (IDH3α) downregulation—a critical metabolic change promoting CAFs activation (transformation of fibroblasts to CAFs). Such a process is associated with a metabolic switch from OXPHOS to aerobic glycolysis [67]. Interestingly, our work revealed that the succinate level is increased in the fibrotic stroma in the luminal A tumors (but not in the luminal B ones) in reference to the extratumoral fibrous connective tissue (Figure 10). However, the positive association between the content of this metabolite and the cancer cell fraction was detected in the latter subtype. The succinate level in the pure cancer tissue in the luminal B tumors, though not reaching a statistical significance, was shown to be higher than in the luminal A ones (Figure 10). A deregulation of the isocitrate dehydrogenase enzyme isoforms 1 (IDH1) and 2 (IDH2) in the epithelial component of breast cancer tumors could be related to these findings. Liu et al. [68] proved that the protein expression levels of IDH1 were gradually decreased during breast cancer progression: from the normal tissue, through ductal carcinoma in situ (DCIS), to invasive cancer; whereas Minemura et al. [69] reported that a high IDH2 status was found to be significantly linked to poorer patient prognosis and was identified as an independent prognostic factor for patients with estrogen-receptor positive status. The analysis of a metabolic reprogramming in lung myo-fibroblasts indicates that accumulation of the TCA intermediates (including succinate) in fibrotic tissue could also be related to the enhanced glycolytic flux [70] and/or glutaminolysis [71]. The latter process was found to be essential in a TGF-β1 (Transforming Growth Factor β1)-mediated differentiation and an activation of lung fibroblasts [71]. Interestingly, Choi et al. reported a high expression of glutaminase in the tumoral stroma characterized by an enhanced glycolytic activity (the reverse Warburg and the mixed phenotypes) [46]. A catabolism of glutamine to glutamate in CaFs is critical for production α-ketoglutarate–a precursor in collagen synthesis—the major protein component within an extracellular space. The increased stromal level of glutamate found in our work may be related to these processes in CAFs. However, the accumulation of this metabolite in the stroma can also result from the enhanced glutaminolysis in cancer cells and a secretion of a portion of glutamate to the tumor microenvironment [72]. According to the working model proposed by Ko, glutamine secreted from autophagic fibroblasts is taken up by cancer cells and increases their mitochondrial activity, whereas ammonia (the byproduct of glutaminolysis) diffuses into the microenvioronment and promotes autophagy and glutamine synthesis in CAFs [73]. This vicious cycle was shown to play an important role in the growth of cancer cells. It is worth mentioning that the amino acid exchange between CAFs and cancer cells (including glutamate) was found to be modulated by extracellular matrix stiffness [74].

Myo-inositol was detected by us to be more abundant in the fibrotic stroma than in the healthy fibrous connective tissue. This compound acts as an osmolyte and can be incorporated into phospholipids. A joint analysis of metabolomics and transcrptomics data revealed that the NMR-visible level of myo-inositol in breast tumors was correlated to the gene ontology terms associated with an extracellular matrix and a cell adhesion [43]. Our results seem to confirm these observations, as no significant relationship between this metabolite level and the cancer cell percentage suggests that this compound is, in fact, related to the metabolism of stroma. Similar results were also obtained for another compound belonging to the inositols family, scyllo-inositol. However, a weak positive correlation between the level of this metabolite and the cancer cell fraction was detected in the grade II/III tumors.

According to [63], the metabolomic analysis of the mammary fat pad composition of Caveolin-1 (-/-) null mice—a model of pure tumor stroma without cancer cells—revealed the up-regulation of over 100 metabolites compared to wild-type tissue. A considerable increase in ascorbic acid (11-fold) was speculated to be a response to oxidative stress. The accumulation of this metabolite in the stromal compartment was also detected in our work. In agreement with our results, Pavlides et al. also detected the increases in glutamate, myo-inositol, glycine, choline, phosphoethanolamine, and hypotaurine (a compound involved in taurine and hypotaurine metabolism) in the stroma of Caveolin-1 (-/-) null mice.

The main strengths of this study include the use of the ^1^H MAS NMR method combined with the direct histopathology examinations and the multivariate classification modelling checked with the univariate linear regression. Such a combination resulted in high classification accuracy. As far as we know, there are still few such works in the literature.

Our study had some limitations, which need to be taken into account when interpreting the data. The analyses are based mainly on invasive ductal carcinoma and invasive lobular carcinoma; other tumor types were not considered. Another drawback is a small number of patients diagnosed with grade I and III tumors. When the types of tissues are taken into account, it was unfortunately not possible to include glandular tissue and necrosis in the analyses. Thus, further studies are needed.

## 5. Conclusions

From the results of our study the following conclusions can be drawn:

1. ^1^H HR-MAS NMR spectroscopy coupled with a multivariate OPLS-DA analysis permitted classification of the cancerous vs. non-cancerous tissues with an accuracy of 87% (sensitivity of 72.2%, specificity of 92.3%);
2. The correlation coefficients between the metabolite levels and cancer cell fraction were found to be grade dependent;
3. The comparison of the tumor purity adjusted levels of the evaluated compounds revealed increased lactate, phosphoethanolamine, taurine, glycine, creatine and glutamate in the grade II/III tumors in comparison to the grade I ones;
4. The analysis of the metabolic composition of intratumoral fibrosis (determined using a linear regression approach) revealed both shared and unique metabolic features observed in the breast tumors of different histological grades (I vs. II/III) in reference to the extratumoral fibrous connective tissue. The shared features include the lactate, glutamate and succinate accumulation within the fibrotic compartment, whereas the increased creatine, phosphoethanolamine, taurine and glycine levels were observed in the stroma of the grade I tumors only;
5. The levels of ascorbate, choline, myo-inositol, and scyllo-inositol were found to be increased in the fibrotic tissue in the grade I tumors and trended towards an increase in the higher-grade (II/III) ones;
6. Although the analysis of the steady-state metabolite levels does not allow for the unambiguous interpretation of the cells interaction within the tumor microenvironment, the results of our study contribute to an understanding of breast tumor molecular heterogeneity which is becoming increasingly important in the era of the introduction of metabolic profiling directly into the surgical theatre.

## Figures and Tables

**Table 1:** The patients’ clinico-pathological characteristics.

Age (Median (Range))	50 (47–78) Years
Tumor type	Invasive carcinoma–32
	Invasive lobular carcinoma–7
Grade	G1–11
	G2–21
	G3–6
	Missing data–1
T-classification	T1–22
	T2–17
N-classification	N0–39 (100%)
Receptor status	ER positive–36 (92.3%)
	PR positive–32 (82.1%)
	HER2 positive–4 (10.3%)
Subtype	Luminal A–22
	Luminal B (HER2 negative/HER2 positive)–11/3
	HER2–1
	TNBC–2

**Table 2:** The tissue contents of the samples collected at the T, TB, N_d<1cm_, N_d=1cm_ and N_d>1cm_ locations.

Excision Area	Total Number of Samples/Number of Samples Containing Cancer Cells	Statistics	Tumor Tissue [%]	Cancer Cells [%]	Intratumoral Fibrosis [%]	Extratumoral Fibrous Connective Tissue [%]	Extratumoral Fatty Tissue [%]	Glandular Tissue [%]	Immune Cells [%]	Necrosis [%]
T	38/36	Median	100	30	70	0	0	0	0	0
		Minimum	0	0	0	0	0	0	0	0
		Maximum	100	75	95	95	45	20	50	30
		1st quartile	95	10	40	0	0	0	0	0
		3rd quartile	100	40	80	0	5	0	0	0
TB	11/8	Median	60	3	30	5	20	0	5	0
		Minimum	0	0	0	0	0	0	0	0
		Maximum	100	30	92	75	60	15	25	20
		1st quartile	5	0	0	0	0	0	0	0
		3rd quartile	100	25	85	40	50	0	5	0
N_d<1cm_	26/9	Median	0	0	0	7.5	30	0	0	0
		Minimum	0	0	0	0	0	0	0	0
		Maximum	100	50	80	90	100	20	5	0
		1st quartile	0	0	0	0	10	0	0	0
		3rd quartile	50	20	25	60	90	2	0	0
N_d=1cm_	30/1	Median	0	0	0	22.5	75	0	0	0
		Minimum	0	0	0	0	0	0	0	0
		Maximum	5	5	0	100	100	20	5	0
		1st quartile	0	0	0	0	40	0	0	0
		3rd quartile	0	0	0	50	95	10	0	0
N_d>1cm_	35/0	Median	0	0	0	0	100	0	0	0
		Minimum	0	0	0	0	0	0	0	0
		Maximum	0	0	0	100	100	10	0	0
		1st quartile	0	0	0	0	60	0	0	0
		3rd quartile	0	0	0	40	100	0	0	0

**Table 3:** The locations of the excisions of the samples and the inclusion criteria for the specimens most representative of a specific tissue type.

Tissue Type	Localization/Number of Samples	Inclusion Criteria
Cancer (C)	T/12 N_d<1cm_/1	Cancer cells ≥ 40%, Fatty tissue ≤ 10%, Glandular tissue ≤ 10%
Intratumoral fibrotic stroma (IF)	T/9 TB/3	Fibrotic stroma ≥ 80% Cancer cells ≤ 15%
Extratumoral fibrous connective tissue at a distance < 1 cm from tumor border (EC, d < 1 cm)	N_d<1cm_/5	Connective tissue ≥ 80%
Extratumoral fibrous connective tissue at a distance ≥ 1 cm from tumor border (EC, d ≥ 1 cm)	N_d=1cm_/4 N_d>1cm_ /5	Connective tissue ≥ 80%
Extratumoral fatty tissue at a distance <1 cm from tumor border (EF, d < 1 cm)	N_d<1cm_/5	Fatty tissue = 100%
Extratumoral fatty tissue at a distance ≥1 cm from tumor border (EF, d ≥ 1 cm)	N_d=1cm_/7 N_d>1cm_/18	Fatty tissue = 100%

## Data Availability

The datasets generated during and/or analyzed during the current study are available from the corresponding author on reasonable request.

## Supplementary Materials

The following supporting information can be downloaded at: https://www.mdpi.com/article/10.3390/cancers15041283/s1, Table S1: The integral regions and the metabolites contributing to these regions in ^1^H CPMG HR-MAS and ^1^H DIFF HR-MAS NMR spectra; Table S2: The relations between the integral intensities in the evaluated spectral regions and fatty tissue content in the samples excised at N_d=1cm_ and N_d>1cm_ locations; Table S3: The relations between the integral intensities of choline containing compounds and fatty tissue content in the samples excised at N_d=1cm_ and N_d>1cm_ locations; Figure S1: H&E staining of the post-HR-MAS NMR samples: cancer tissue (a), intratumoral fibrosis (b), extratumoral fibrous connective tissue (c); Figure S2: The OPLS-DA model 1: the predicted scores plot colored according to the contents of an intratumoral fibrosis (a), an extratumoral connective tissue (b) and an extratumoral fatty tissue (c); Figure S3: The relationship between the predicted analysis power and sample size estimated retrospectively based on the dataset used for OPLS-DA model 1 development; Figure S4: The OPLS-DA model 2: the predicted scores plot colored according to the content of an intratumoral fibrosis (a), an extratumoral connective tissue (b) and an extratumoral fatty tissue (c); Figure S5: The relationship between the predicted analysis power and sample size estimated retrospectively based on the dataset used for OPLS-DA model 2 development; Figure S6: The OPLS-DA model 3 (1 predictive + 1 orthogonal latent variable, R2X = 73.6%, R2Y = 65.8%, Q2 = 58.6%, CV-ANOVA *p* value = 8.02 × 10^−13^): the cross-validated scores plot colored according to the sample category (cancerous vs normal) (a), content of cancer cells (b), content of an intratumoral fibrosis (c) content of an extratumoral connective tissue (d), content of glandular tissue (e), content of an extratumoral fatty tissue (f), excision area (g); Figure S7: The relationships between choline (a), ascorbate (b), scyllo-inositol (c) myo-inositol and a cancer cell fraction.
