# Supplementary material for: The Relationship between Histological Composition and Metabolic Profile in Breast Tumors and Peritumoral Tissue Determined with 1H HR-MAS NMR Spectroscopy
Source: Cancers (Basel). 2023 Feb 17;15(4):1283. doi: 10.3390/cancers15041283 (PMC9954108; doi:10.3390/cancers15041283)
Supplement: Supplementary file 1 [file cancers-15-01283-s001.zip › cancers-2192073-supplementary.pdf]

Supplementary Materials: The relationship between histological composition and metabolic profiles in breast tumors and peritumoral tissue determined with ^{1}H HR MAS NMR spectroscopy

Agnieszka Skorupa, Mateusz Ciszek, Maria Turska-d'Amico, Ewa Stobiecka, Ewa Chmielik, Ryszard Szumniak, Andrea d'Amico, Łukasz Boguszewicz, Maria Sokół

Table S1. The integral regions and the metabolites contributing to these regions in ^{1}H CPMG HR MAS and ^{1}H DIFF HR MAS NMR spectra. The major metabolites used for presentation of results are underscored.

**Spectrum**	**The integral regions**	**Metabolite, chemical shift (multiplicity)**
**CPMG**	4.67– 4.61 ppm	glucose, 4.64 ppm (d)
	4.53 – 4.50 ppm	ascorbate, 4.51 ppm (d)
	4.15- 4.07 ppm	lactate, 4.11 ppm (q); proline, 4.12 ppm (dd)
	4.07- 4.02 ppm	myo-inositol, 4.05 ppm (t); choline, 4.05 ppm (m)
	4.00 - 3.94 ppm	phosphoethanolamine, 3.97 ppm (m); serine, 3.96 ppm (m)
	3.80-3.73 ppm*	amino acids (glutamine, glutamate, alanine, aspartate, asparagine, lysine), 3.77 ppm (m); glucose, 3.76 ppm (dd)
	3.44 -3.38 ppm	taurine, 3.41 ppm (t); glucose, 3.39 ppm (t)
	3.35 – 3.32 ppm	scyllo-inositol, 3.33 ppm (s); proline, 3.32 (m)
	3.04-3.00 ppm	creatine, 3.02 ppm (s); lysine 3.02 ppm (t)
	2.37-2.30 ppm	glutamate, 2.35 ppm (m); proline, 2.34 ppm (m)
	2.47-2.41 ppm	glutamine, 2.44 ppm (m)
	2.41-2.38 ppm	succinate, 2.40 ppm (s)
	The regions determined using line fitting	choline, 3.19 ppm (s)
		phosphocholine, 3.21 ppm (s)
		glycerophosphocholine, 3.22 ppm (s)
**DIFF**	0.95-0.8 ppm	lipid ($-CH_2-C\mathbf{H}_3$), 0.9 ppm
	1.45-1.15 ppm	lipid [$-(C\mathbf{H}_2)_n-$], 1.3 ppm
	1.69-1.46 ppm	lipid [$-C\mathbf{H}_2-CH_2-COO$], 1.6 ppm
	2.14-1.88 ppm	lipid [$-C\mathbf{H}_2-CH=CH-$], 2.03 ppm
	2.40-2.14 ppm	lipid [$-CH_2-C\mathbf{H}_2-COO$], 2.25 ppm
	3-2.62 ppm	lipid, [$-CH=CH-C\mathbf{H}_2-CH=CH-$], 2.78 ppm
	4.18-3.94 ppm	lipid, [$C\mathbf{H}_2-O-CO$], 4.10 ppm
	4.50-4.18 ppm	lipid, [$C\mathbf{H}_2-O-CO$], 4.30 ppm

s- singlet, d-doublet, dd – doublet of doublets, t- triplet, q-quartet, m –multiplet

* no major compound identified

Table S2. The relations between the integral intensities in the evaluated spectral regions and fatty tissue content in the samples excised at $N_{d=1\ cm}$ and $N_{d>1\ cm}$ locations.

**Spectral region (major contributing metabolite, chemical shift)**	**The relation between the integral intensities in the evaluated spectral regions (y) and fatty tissue content (x)**
4.67– 4.61 (Glucose, 4.64 ppm)	y = 97179.2631 - 565.7017*x;<br>r = -0.6405; p = 0.00000; r^2 = 0.4102
4.53 – 4.50 (Ascorbate, 4.51 ppm)	y = 39556.0249 - 196.7389*x;<br>r = -0.5921; p = 0.00000; r^2 = 0.3506
4.15-4.07 (Lactate, 4.11 ppm)	y = 2.662E5 - 1833.4274*x;<br>r = -0.5514; p = 0.00000; r^2 = 0.3040
4.07-4.02 (Myo-inositol, 4.05 ppm)	y = 1.445E5 - 330.3301*x;<br>r = -0.3516; p = 0.0044; r^2 = 0.1236
4.00-3.94 (Phosphoethanolamine, 3.97 ppm)	y = 1.55E5 - 293.8152*x;<br>r = -0.2425; p = 0.0535; r^2 = 0.0588
3.77 ppm (Amino acids)	y = 3.5605E5 - 2055.9018*x;<br>r = -0.5914; p = 0.00000; r^2 = 0.3498
3.56 -3.54 ppm (Glycine, 3.55 ppm)	y = 1.5426E5 - 984.9302*x;<br>r = -0.5500; p = 0.00000; r^2 = 0.3025
3.44 -3.38 ppm (Taurine, 3.41 ppm)	y = 3.6712E5 - 2507.054*x;<br>r = -0.6145; p = 0.00000; r^2 = 0.3776
3.35 – 3.32 ppm (Scyllo-inositol, 3.33 ppm)	y = 39321.9528 - 93.8116*x;<br>r = -0.2600; p = 0.0380; r^2 = 0.0676
3.04-3.00 ppm (Creatine, 3.02 ppm)	y = 1.0713E5 - 255.9473*x;<br>r = -0.3533; p = 0.0042; r^2 = 0.1248

r- Pearson correlation coefficient

Table S3. The relations between the integral intensities of choline containing compounds and fatty tissue content in the samples excised at $N_{d=1\ cm}$ and $N_{d>1\ cm}$ locations.

**Metabolite**	**The relation between the integral intensities in the evaluated spectral regions (y) and fatty tissue content (x)**
Choline (3.20 ppm)	y = 3.334E5 - 2117.8647*x; r = -0.5951; p = 0.00000; r^2 = 0.3541
Phosphocholine (3.21 ppm)	y = 1.1514E5 - 904.7418*x; r = -0.3389; p = 0.0062; r^2 = 0.1149
Glycerophosphocholine (3.22 ppm)	y = 2.12E5 - 686.4217*x; r = -0.3553; p = 0.0040; r^2 = 0.1262

r- Pearson correlation coefficient

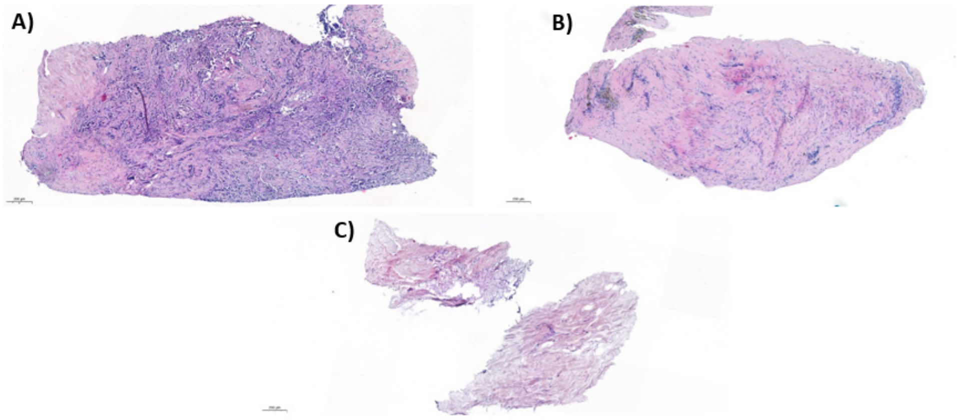

Figure S1. H&E staining of the post-HR MAS NMR samples: cancer tissue (a), intratumoral fibrosis (b), extratumoral fibrous connective tissue (c).

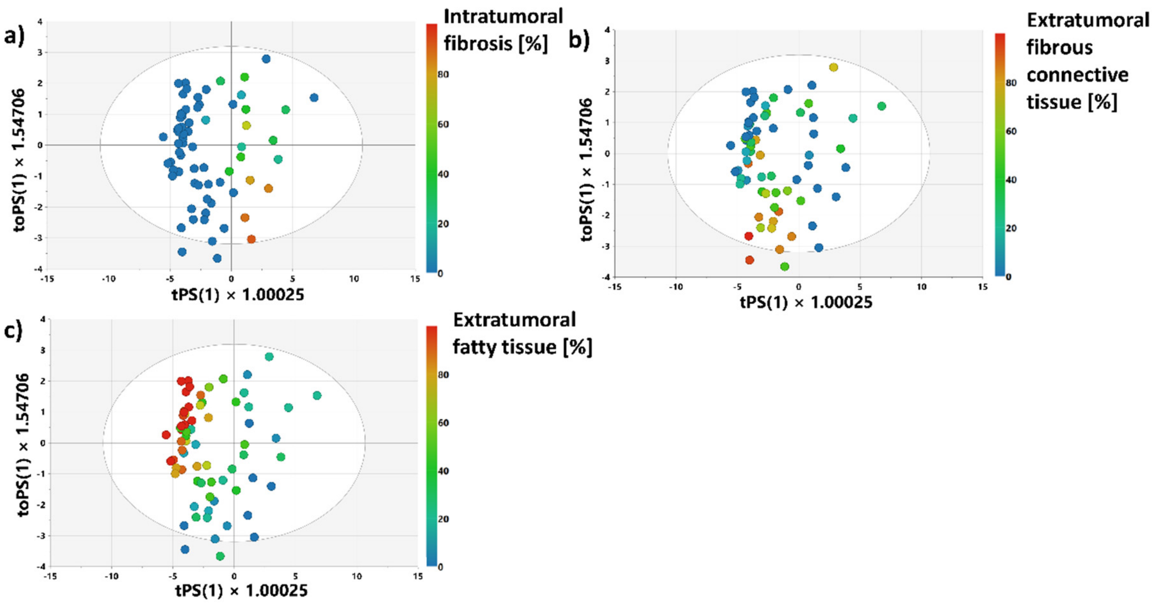

Figure S2. The OPLS-DA model 1: the predicted scores plot colored according to the contents of an intratumoral fibrosis (a), an extratumoral connective tissue (b) and an extratumoral fatty tissue (c). The predicted scores for the predictive component are denoted as tPS(1), while those for the orthogonal one – as toPS(1).

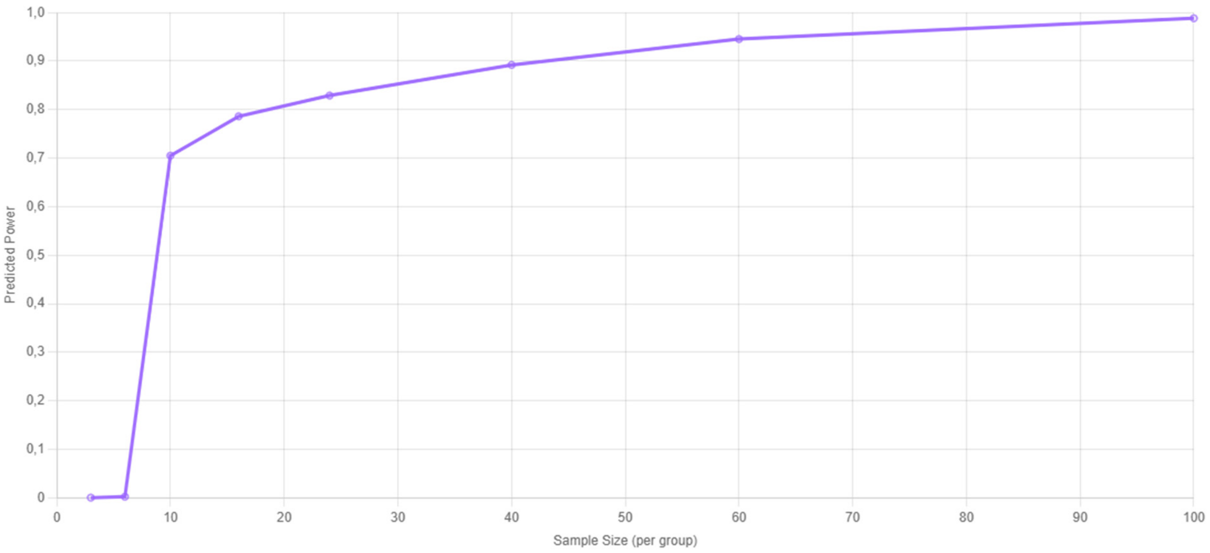

Figure S3. The relationship between the predicted analysis power and sample size (FDR adjusted p value = 0.001) estimated retrospectively based on the dataset used for OPLS-DA model 1 development.

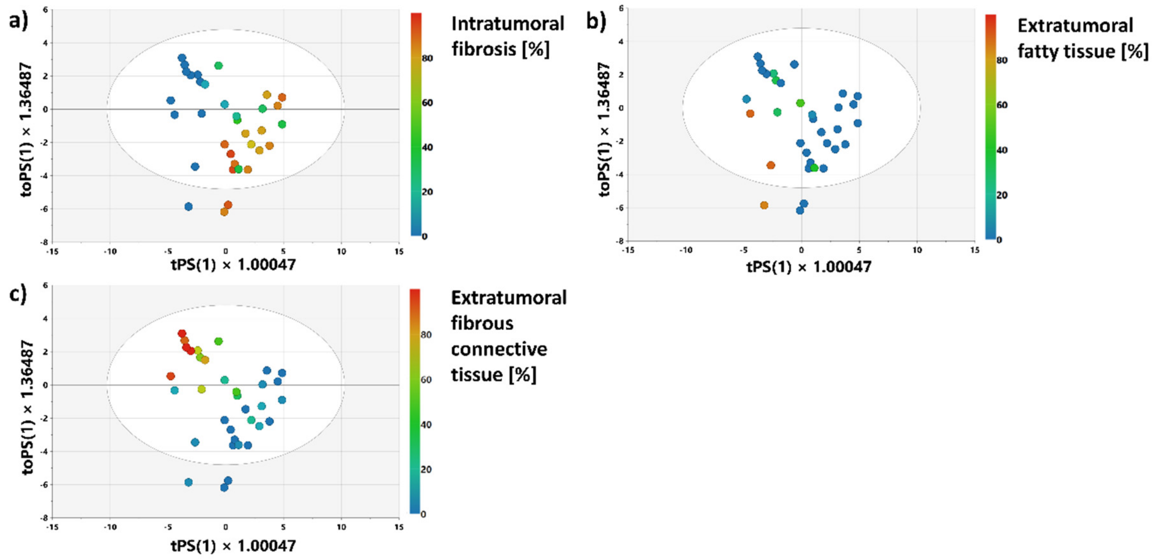

Figure S4. The OPLS-DA model 2: the predicted scores plot colored according to the content of an intratumoral fibrosis (a), an extratumoral connective tissue (b) and an extratumoral fatty tissue (c). The predicted scores for the predictive component are denoted as tPS(1), while those for the orthogonal one – as toPS(1).

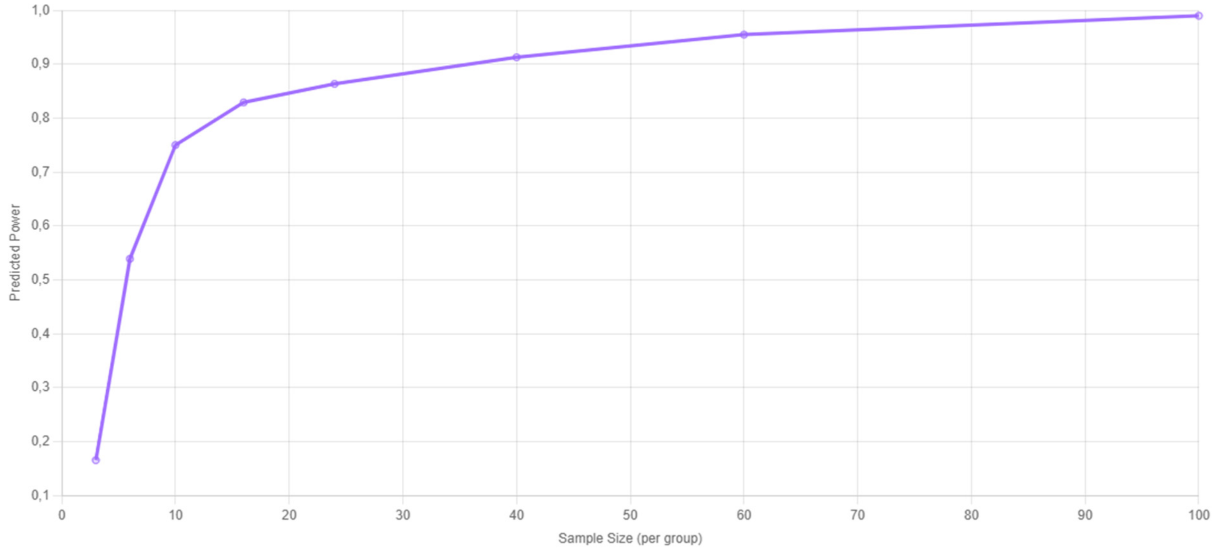

Figure S5. The relationship between the predicted analysis power and sample size (FDR adjusted p value = 0.001) estimated retrospectively based on the dataset used for OPLS-DA model 2 development.

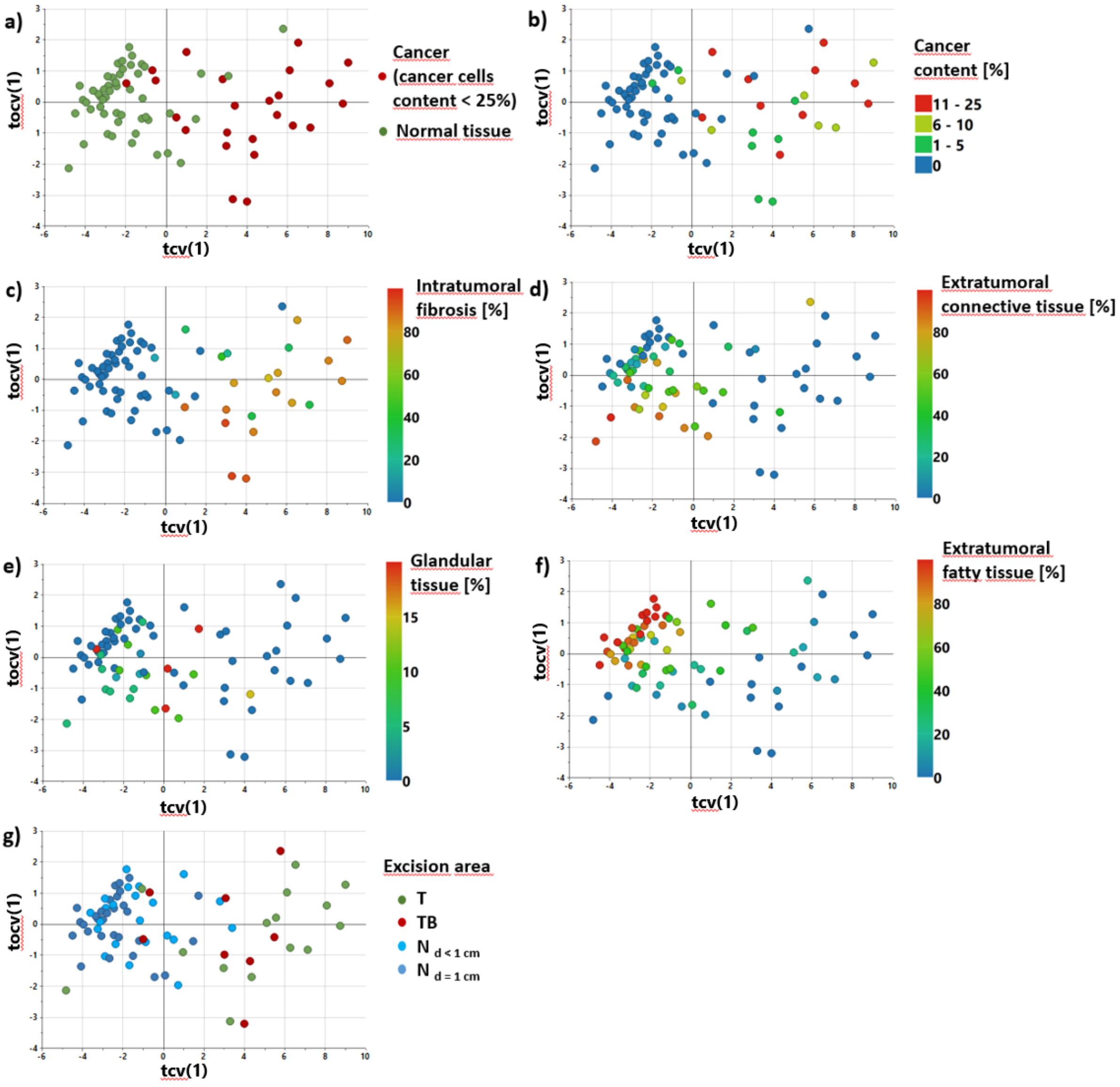

Figure S6. The OPLS-DA model 3 (1 predictive + 1 orthogonal latent variable, R2X = 73.6%, R2Y = 65.8%, Q2 = 58.6%, CV-ANOVA p value = 8.02 x 10^{-13}): the cross-validated scores plot colored according to the sample category (cancerous vs normal) (a), content of cancer cells (b), content of an intratumoral fibrosis (c) content of an extratumoral connective tissue (d), content of glandular tissue (e), content of an extratumoral fatty tissue (f), excision area (g). The cross-validated scores for the predictive component are denoted as tcv(1), while those for the orthogonal one – as tocv(1).

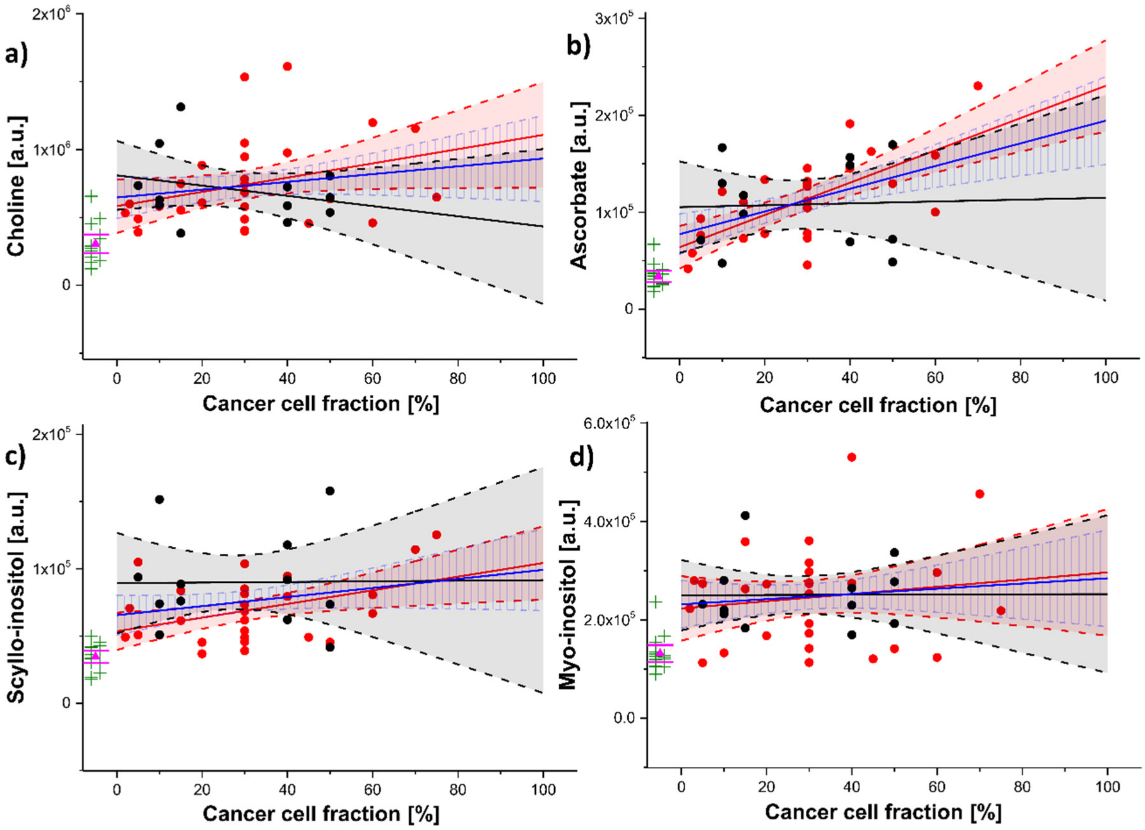

● Samples containing mainly intratumoral fibrosis and cancer cells (grade I)

● Samples containing mainly intratumoral fibrosis and cancer cells (grade II/III)

Linear regression fits with 90% confidence interval bands: analysis of tumors of all grades; analysis of grade I tumors; analysis of grade II/III tumors

+ Histologically non-transformed samples containing mainly extratumoral connective tissue (≥ 80%) (the samples collected at the distance ≥ 1 cm from the tumor border are artificially shifted to -6 % cancer content, while the samples excised at the distance < 1 cm – to -4 % cancer content for visualization purposes)

▲ Mean metabolite level for the samples containing mainly extratumoral connective tissue and its 90% confidence interval

Figure S7. The relationships between choline (a), ascorbate (b), scyllo-inositol (c) myo-inositol and a cancer cells fraction. The linear fits (blue solid lines – the analysis of the tumors of all grades, black solid lines – the analysis of the grade I tumors, red solid line – the analysis of the grade II/III tumors) with 90% confidence bands (blue dashed lines – the analysis of the tumors of all grades, black dashed lines – the analysis of the grade I tumors, red dashed lines – the analysis of the grade II/III tumors) were calculated for the samples containing mainly intratumoral fibrosis and cancer cells. The levels of the metabolites corresponding to pure intratumoral fibrosis and pure cancer are determined from the extrapolation of the regression lines to 0% and 100% cancer content. Additionally, the metabolite levels measured in the samples representative of extratumoral fibrous connective tissue (green crosses) are presented. The pink triangle and pink lines indicate the mean metabolite levels obtained for these samples and its 90% confidence interval.
